# Dhh1 promotes autophagy-related protein translation during nitrogen starvation

**DOI:** 10.1371/journal.pbio.3000219

**Published:** 2019-04-11

**Authors:** Xu Liu, Zhiyuan Yao, Meiyan Jin, Sim Namkoong, Zhangyuan Yin, Jun Hee Lee, Daniel J. Klionsky

**Affiliations:** 1 Life Sciences Institute, and the Department of Molecular, Cellular and Developmental Biology, University of Michigan, Ann Arbor, Michigan, United States of America; 2 Department of Molecular and Integrative Physiology, University of Michigan, Ann Arbor, Michigan, United States of America; New York University School of Medicine, UNITED STATES

## Abstract

Macroautophagy (hereafter autophagy) is a well-conserved cellular process through which cytoplasmic components are delivered to the vacuole/lysosome for degradation and recycling. Studies have revealed the molecular mechanism of transcriptional regulation of autophagy-related (*ATG*) genes upon nutrient deprivation. However, little is known about their translational regulation. Here, we found that Dhh1, a DExD/H-box RNA helicase, is required for efficient translation of Atg1 and Atg13, two proteins essential for autophagy induction. Dhh1 directly associates with *ATG1* and *ATG13* mRNAs under nitrogen-starvation conditions. The structured regions shortly after the start codons of the two *ATG* mRNAs are necessary for their translational regulation by Dhh1. Both the RNA-binding ability and helicase activity of Dhh1 are indispensable to promote Atg1 translation and autophagy. Moreover, eukaryotic translation initiation factor 4E (EIF4E)-associated protein 1 (Eap1), a target of rapamycin (TOR)-regulated EIF4E binding protein, physically interacts with Dhh1 after nitrogen starvation and facilitates the translation of Atg1 and Atg13. These results suggest a model for how some *ATG* genes bypass the general translational suppression that occurs during nitrogen starvation to maintain a proper level of autophagy.

## Abstract 2

The precise regulation of autophagy is critical to maintaining proper cell physiology. This study shows that translational regulation involving the RNA helicase Dhh1 plays an important role in controlling the level of the Atg1 kinase, a key factor in autophagy induction.

## Introduction

Autophagy is a tightly controlled cellular process by which cytosolic proteins, protein aggregates, damaged or surplus organelles, and invading pathogens are sequestered within a double-membrane vesicle (the autophagosome) and then delivered to the vacuole/lysosome for degradation and recycling [1,2]. Autophagy is highly conserved among eukaryotes. Malfunction of autophagy has been associated with many human diseases, including cancer, myopathies, liver, heart and lung disease, and neurodegeneration [3–6].

Studies in yeasts have identified more than 40 autophagy-related (*ATG*) genes involved in mediating autophagy [7], and many of the corresponding gene products have homologs or functional counterparts in higher eukaryotes [2]. The expression levels of *ATG* genes are important for maintaining proper levels of autophagy activity [8,9]. Expression of most *ATG* genes is up-regulated under autophagy-inducing conditions such as nutrient deprivation [10]. The transcriptional regulation of *ATG* genes has been extensively investigated, and an increasing number of transcriptional activators and repressors involved in autophagy regulation are being characterized in both yeast and mammals [8,10–14].

Two recent studies from our lab and collaborators showed that under nutrient-rich conditions, *ATG* mRNAs are down-regulated at the posttranscriptional level [15,16]. The main mRNA decapping 2 (Dcp2) mediates the decapping of almost all major *ATG* mRNAs, whereas the RNA exoribonuclease 1 (Xrn1) is responsible for the degradation of some of them. A subset of *ATG* mRNAs is conveyed to the decapping machinery by Dhh1, a DExD/H-box RNA helicase. These studies demonstrated that *ATG* mRNAs are degraded by decapping-mediated mRNA decay so that their expression is restricted to maintain autophagy activity at a basal level under nutrient-rich conditions. Our lab recently also reported that the conserved Pat1–Lsm complex protects some *ATG* mRNAs from exosome-dependent 3′-5′ degradation under nitrogen-starvation conditions. This allows stabilization of these *ATG* mRNAs and efficient expression to induce autophagy during nitrogen starvation [17].

However, it is not well known whether and how the expression of *ATG* genes is translationally regulated when nutrients are limited. Under nutrient-deprivation conditions, the translation of most genes across the genome is down-regulated, whereas expression of most *ATG* genes is up-regulated and maintained at relatively high levels to support the increased demands of autophagy activity. This suggests that there is likely to be a specialized regulation mechanism through which the *ATG* mRNAs escape the general translation inhibition when nutrients are depleted, conditions under which autophagy becomes essential.

In this study, we found that in contrast to its role as an autophagy inhibitor when nutrients are rich, Dhh1 facilitates translation of *ATG1* and *ATG13* mRNAs under nitrogen-starvation conditions. Dhh1 associates with *ATG1* and *ATG13* mRNAs under this condition. The helicase activity of Dhh1 is necessary to drive Atg protein translation, and the structured regions shortly after the start codons of *ATG1* and *ATG13* mRNAs are required for their Dhh1-dependent translation. Moreover, under nitrogen-starvation conditions when target of rapamycin 1 (TOR1) activity is inhibited, Dhh1 physically interacts with eukaryotic translation initiation factor 4E (EIF4E)-associated protein 1 (Eap1), an EIF4E binding protein (EIF4EBP), to associate with the translation initiation complex. Despite its well-known roles as a repressor of cap-dependent translation, Eap1 promotes Atg1 and Atg13 translation under nitrogen-starvation conditions. Finally, we show that DEAD-box helicase 6 (DDX6), the mammalian homolog of Dhh1, is required for translational up-regulation of autophagy related 16 like 1 (*ATG16L1*) during amino acid starvation in human embryonic kidney 293A (HEK293A) cells.

## Results

### Dhh1 positively regulates autophagy under nitrogen-starvation conditions

**Fig 1:**
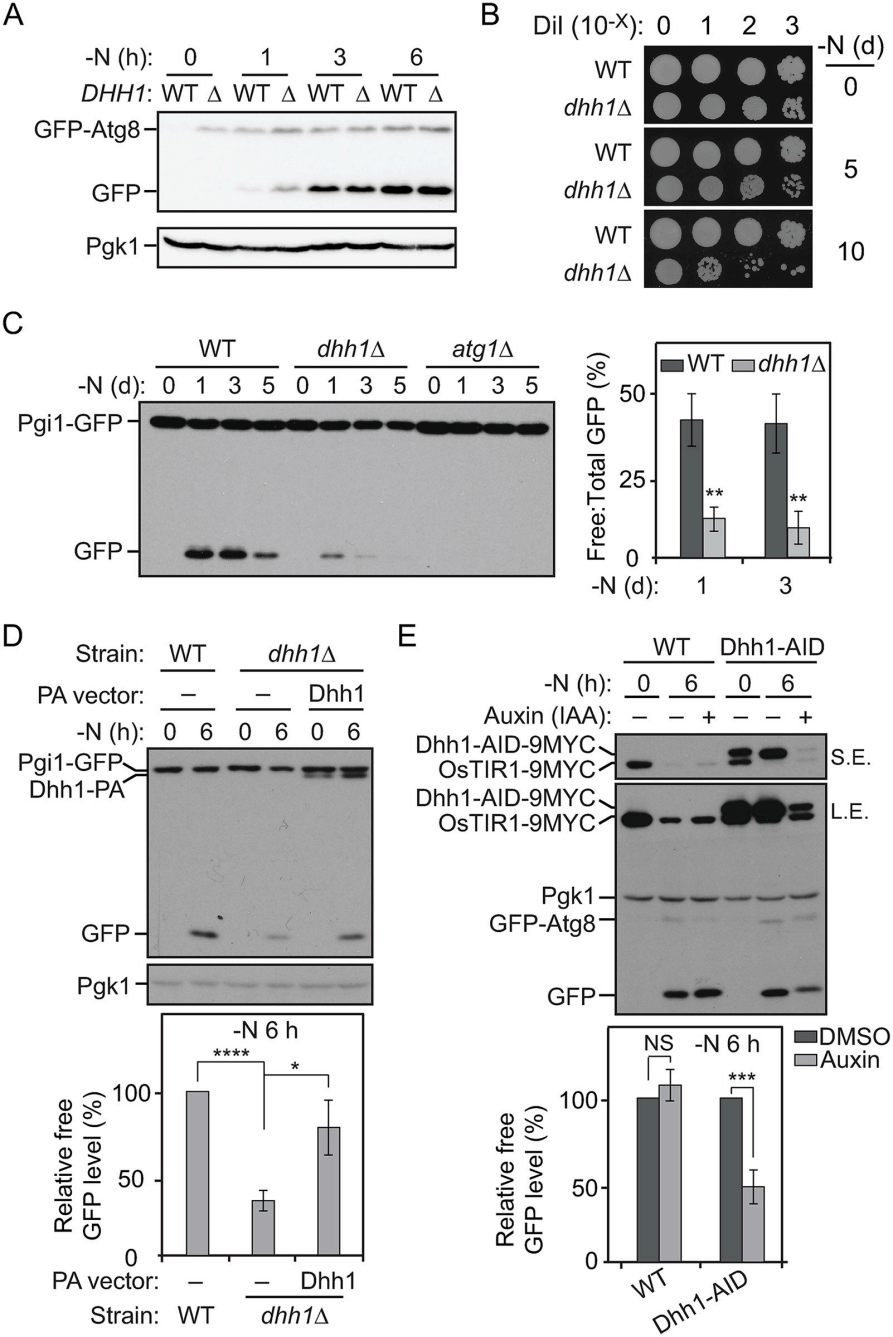
Dhh1 positively regulates autophagy under nitrogen-starvation conditions. (A) GFP–Atg8 (ZYY101) and GFP–Atg8 *dhh1Δ* (ZYY201) cells were grown in YPD to mid-log phase (-N, 0 h) and then shifted to SD-N for 1, 3, and 6 h. Cell lysates were prepared, subjected to SDS-PAGE, and analyzed by western blot. Pgk1 was a loading control. The expression of GFP–Atg8 was driven by its endogenous promoter. (B) WT (SEY6210) and *dhh1Δ* (XLY301) cells were grown in YPD to mid-log phase (-N, 0 d) and then shifted to SD-N for 5 and 10 d. The indicated dilutions of cells were plated on YPD plates and grown for 2 d. (C) Pgi1–GFP (XLY306), Pgi1–GFP *atg1Δ* (XLY307), and Pgi1–GFP *dhh1Δ* (XLY308) cells were grown in YPD to mid-log phase (-N, 0 d) and then shifted to SD-N for 1, 3, and 5 d. Cell lysates were prepared, subjected to SDS-PAGE, and analyzed by western blot. The ratio of free GFP to total GFP (free GFP + Pgi1–GFP) was quantified. Average values ± s.d. of *n* = 3 independent experiments are shown as indicated. **p < 0.01. (D) The Pgi1–GFP strain with empty vector (XLY329), the Pgi1–GFP *dhh1Δ* strain with either empty vector (XLY331), or a plasmid expressing Dhh1^WT^–PA (XLY333) were grown in YPD to mid-log phase (-N, 0 h) and then shifted to SD-N for 6 h. Cell lysates were prepared, subjected to SDS-PAGE, and analyzed by western blot. For quantification, the level of free GFP was first normalized to the loading control Pgk1. The values of the other samples were then normalized to that in WT cells. Average values ± s.d. of *n* = 3 independent experiments are shown as indicated. *p < 0.05. ****p < 0.0001. (E) GFP–Atg8 (XLY339) and GFP–Atg8 Dhh1–AID (XLY341) cells were grown in YPD to mid-log phase and treated with either DMSO or 300 μM IAA for 30 min. They were then shifted to SD-N for 6 h in the presence of either DMSO or IAA. Cell lysates were prepared, subjected to SDS-PAGE, and analyzed by western blot. The expression of GFP–Atg8 was driven by its endogenous promoter. For quantification, the level of free GFP was first normalized to the loading control Pgk1. The values of the IAA-treated samples were then normalized to that in DMSO (IAA “-”)-treated samples. Average values ± s.d. of *n* = 3 independent experiments are shown as indicated. ***p < 0.001. (See also S1 Fig and S1 Table; raw numerical values are shown in S1 Data). AID, auxin-inducible degron; Atg8, autophagy-related 8; DMSO, dimethyl sulfoxide; IAA, indole-3-acetic acid; L.E., long exposure; NS, not significant; OsTIR1, *Oryza sativa* transport inhibitor response 1; PA, protein A; Pgi1, phosphoglucoisomerase 1; Pgk1, 3-phosphoglycerate kinase 1; SD-N, synthetic minimal medium lacking nitrogen; S.E., short exposure; WT, wild type; YPD, yeast extract–peptone–dextrose.

The DExD/H-box-containing RCK family RNA helicases are highly conserved in eukaryotes [18]. These proteins play important roles in regulating mRNA degradation, storage, and translation. Recently, others and we have shown that under nutrient-rich conditions, Dhh1—a DExD/H-box protein—targets several *ATG* mRNAs, most significantly *ATG8* transcripts, for degradation through the Dcp2-mediated mRNA decapping pathway to restrict autophagy activity [16]. Consistent with this observation, deletion of the *DHH1* gene in yeast cells led to elevated *ATG8* expression and autophagy activity levels under both nutrient-rich and short-term (0–3 h) nitrogen-starvation conditions (Fig 1A). We noticed that after relatively longer periods of starvation (i.e., 6 h or longer), the Atg8 protein level and the autophagy activity in the *dhh1Δ* cells were not significantly different from those of wild-type (WT) cells, suggesting that the inhibitory effects of Dhh1 on *ATG8* expression and autophagy were released after prolonged nitrogen starvation (Fig 1A).

However, the *dhh1Δ* cells displayed much lower viability compared to the WT cells after several days of nitrogen starvation (Fig 1B). An obvious question then is what contributes to the low viability of the *dhh1Δ* cells after prolonged nitrogen starvation and whether autophagy still plays a role. To answer this, we needed to test autophagy activity in the *dhh1Δ* cells under conditions of prolonged nitrogen starvation. However, the commonly used autophagy flux assays in yeast [19], such as the green fluorescent protein (GFP)–Atg8 processing and Pho8Δ60 assays, are not suitable for this purpose, because the autophagy activity in the WT cells tested with these assays usually becomes saturated after 4 to 6 h of nitrogen starvation. Thus, we developed a phosphoglucoisomerase 1 (Pgi1)–GFP-processing assay to quantitatively monitor autophagy activity during prolonged nitrogen starvation. Pgi1 is a long-lived cytosolic protein that has a relatively stable expression even upon prolonged nitrogen-starvation conditions. As with most cytosolic proteins, we expect that, upon starvation, the cytosolic Pgi1–GFP fusion protein is delivered to the vacuole for degradation through nonselective autophagy. The GFP moiety is relatively resistant to vacuolar degradation [20]; thus, consistent with our prediction, we observed an accumulation of the free GFP band in the WT cell lysates by western blot at 1 d after autophagy induction (Fig 1C). There was no processing of Pgi1–GFP in the *atg1Δ* cells that are defective for autophagy, demonstrating that the generation of free GFP was dependent on autophagic degradation of the chimera. In the *dhh1Δ* cells, a substantially reduced amount of free GFP was detected starting at 1 d after nitrogen starvation compared to the WT cells, suggesting that long-term autophagy was impaired in this mutant (Fig 1C and S1A Fig). Similar results were observed when we examined the processing of another long-lived cytosolic fusion protein, fructose-1,6-biphosphate aldolase 1 (Fba1)–GFP (S1B Fig). These data suggest that Dhh1 switches its role as a positive regulator of autophagy after prolonged starvation conditions. The decreased cell survival in the *dhh1Δ* cells after prolonged nitrogen starvation may be due to diminished autophagy activity in the mutant.

Moreover, the *dhh1Δ* cells already showed significant autophagy defects after 6 h of nitrogen starvation based on the Pgi1–GFP processing assay (Fig 1D). Plasmid-based expression of Dhh1–protein A (PA) in the *dhh1Δ* cells significantly suppressed the autophagy defects in the mutant (Fig 1D). These data suggest that Dhh1 positively regulates autophagy under both short and prolonged nitrogen-starvation conditions, the former being in apparent contradiction with the GFP–Atg8 data.

The observation of decreased Pgi1–GFP processing in the *dhh1Δ* strain under both short- and long-term starvation led us to wonder whether the accumulated higher GFP–Atg8 expression under nutrient-rich conditions seen in the *dhh1Δ* cells (Fig 1A; [16]) led to an inaccurate measure of autophagy activity based on the GFP–Atg8-processing assay following a shift to nitrogen-starvation conditions. That is, *dhh1Δ* cells express higher levels of several *ATG* genes in nutrient-rich conditions, and the higher levels of the corresponding proteins lead to an apparent increase in autophagy activity shortly after shifting cells to starvation conditions. Thus, examining the constitutive null mutant could temporarily mask any effects due to the absence of Dhh1 shortly after autophagy induction.

To exclude the effects of Dhh1 on autophagy under nutrient-rich conditions, we took advantage of the auxin-inducible degron (AID) system to conditionally knock down *DHH1* expression under starvation conditions. This system is well established in yeast cells to temporally control gene expression by mediating proteasomal degradation of targeted proteins in an auxin-dependent manner [21]. To induce efficient and fast degradation of Dhh1–AID, we pretreated the cells with 300 μM indole-3-acetic acid (IAA) for 30 min under nutrient-rich conditions. The cells were then shifted to nitrogen-starvation medium, and continued IAA treatment in this condition was sufficient to maintain Dhh1–AID protein at a minimal level (Fig 1E). Compared to the cells treated with dimethyl sulfoxide (DMSO), we observed lower autophagy activity after 6-h nitrogen starvation based on the GFP–Atg8 processing assay (Fig 1E). As a control, IAA treatment in the WT cells did not affect autophagy activity (Fig 1E). Our observations collectively suggest that Dhh1 is a bidirectional regulator of autophagy, whose role is controlled by nutrient conditions. Dhh1 acts as a negative regulator to direct some *ATG* mRNAs for degradation under nutrient-rich conditions [16]. Here, our data imply a newly discovered role for Dhh1 to positively regulate autophagy under nitrogen-starvation conditions.

### Dhh1 promotes the translation of Atg1 and Atg13 under nitrogen-starvation conditions

Next, we decided to determine how Dhh1 promotes autophagy during nitrogen starvation. A recent genome-wide study suggested that Dhh1 is able to both suppress and promote mRNA translation [22]. However, this study was performed under nutrient-rich conditions when autophagy is not induced, and it did not identify any *ATG* mRNAs within the core autophagy molecular machinery being directly translationally regulated by Dhh1. Accordingly, we specifically asked whether Dhh1 regulates mRNA translation under nitrogen-starvation conditions and whether any *ATG* mRNAs are direct targets of Dhh1.

The previous study [22] suggested that a feature of the mRNAs translationally activated by Dhh1 is that the nucleotides at positions 50–120 after the start codons of these mRNAs show a high intrinsic tendency to form secondary structures. We used Structural Profile Assignment of RNA Coding Sequences (SPARCS) [23], a program to analyze structured regions in mRNA coding sequences, to predict if any of the *ATG* mRNAs are potential targets of Dhh1. Out of the 18 *ATG* mRNAs that constitute the core autophagy molecular machinery analyzed, only *ATG1* and *ATG13* mRNAs showed potential structured regions in the open reading frames (ORFs) shortly after the start codons (S2 Table and S2A Fig). Atg1 and Atg13 proteins are the main components of a kinase complex essential for sensing upstream nutrient signals and initiating the autophagy process.

**Fig 2:**
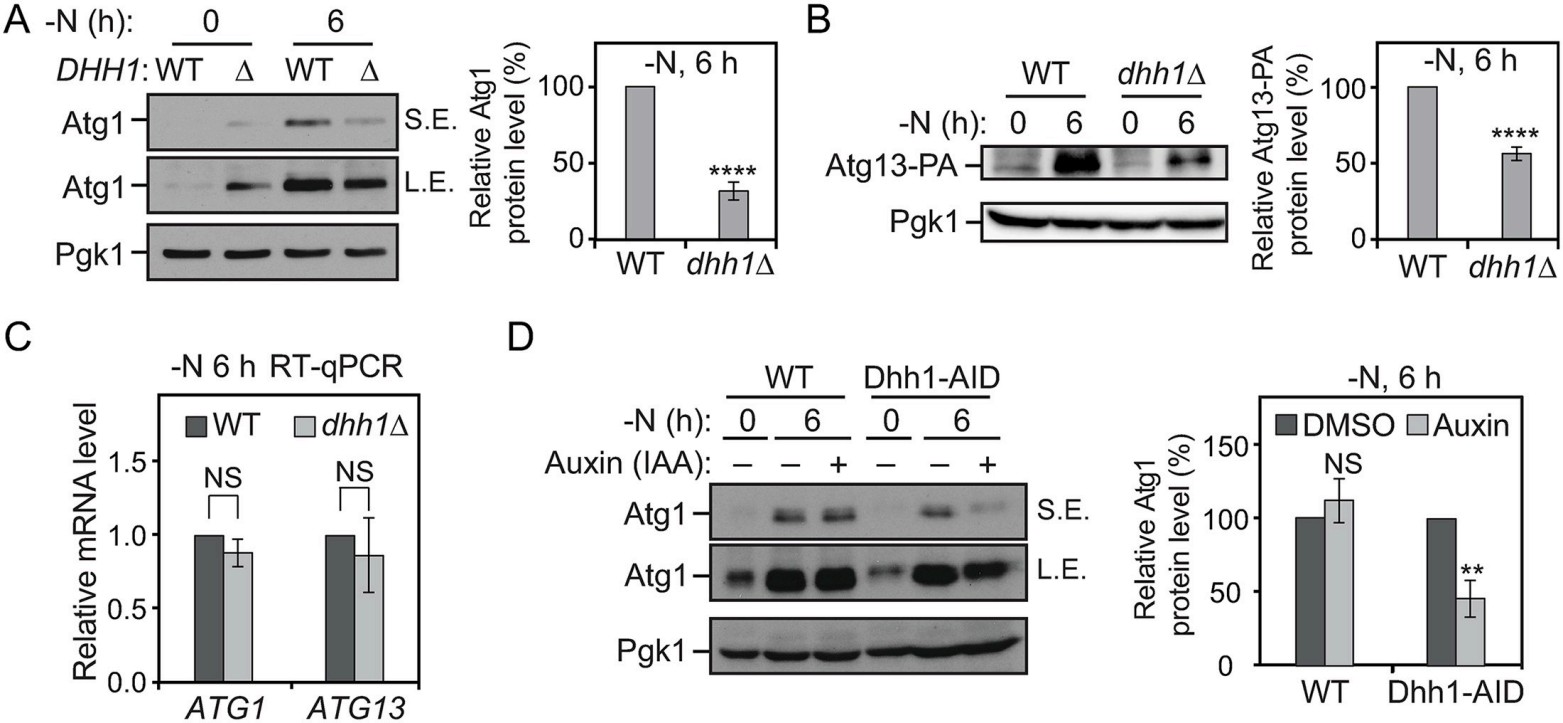
Dhh1 promotes the translation of Atg1 and Atg13 under nitrogen-starvation conditions. (A) WT (SEY6210) and *dhh1Δ* (XLY301) cells were grown in YPD to mid-log phase (-N, 0 h) and then shifted to SD-N for 6 h. Cell lysates were prepared, subjected to SDS-PAGE, and analyzed by western blot. For quantification of Atg1 protein level in cells after prolonged nitrogen starvation, the Atg1 protein level was first normalized to the loading control, Pgk1. The values of the *dhh1Δ* mutant samples were then normalized to that in WT cells. Average values ± s.d. of *n* = 3 independent experiments are shown as indicated. ****p < 0.0001. (B) Atg13–PA (ZYY202) and Atg13–PA *dhh1Δ* (ZYY203) cells were grown in YPD to mid-log phase (-N, 0 h) and then shifted to SD-N for 6 h. Cell lysates were prepared, subjected to SDS-PAGE, and analyzed by western blot. The quantification of Atg13 protein level was conducted as indicated in (A). The 5′-UTR and 3′-UTR of *ATG13* in these strains were not changed. ****p < 0.0001. (C) Atg13–PA and Atg13–PA *dhh1Δ* cells were grown in YPD to mid-log phase (-N, 0 h) and then shifted to SD-N for 6 h. Total RNA for each sample was extracted, and the mRNA levels were quantified by RT-qPCR. The mRNA levels of the samples were first normalized to the level of the reference gene *TAF10*. Then, individual *ATG1* and *ATG13* mRNA levels were normalized to the mRNA level of the corresponding gene in WT cells. Error bars represent the SD of three independent experiments. (D) WT (XLY338) and Dhh1–AID (XLY340) cells were grown in YPD to mid-log phase and treated with either DMSO or 300 μM IAA for 30 min. They were then shifted to SD-N for 6 h in the presence of either DMSO or IAA. Cell lysates were prepared, subjected to SDS-PAGE, and analyzed by western blot. For quantification, the Atg1 protein levels were first normalized to the loading control, Pgk1. The values of the IAA-treated samples were then normalized to that in DMSO (IAA “-”)-treated samples. Average values ± s.d. of *n* = 3 independent experiments are shown as indicated. **p < 0.01. (See also S2 and S3 Figs; raw numerical values are shown in S1 Data). Atg, autophagy-related; DMSO, dimethyl sulfoxide; IAA, indoe-3-acetic acid; L.E., long exposure; NS, not significant; PA, protein A; Pgk1, 3-phosphoglycerate kinase 1; RT-qPCR, quantitative reverse transcription PCR; SD-N, synthetic minimal medium lacking nitrogen; S.E, short exposure; *TAF10*, TATA binding protein-associated factor 10; WT, wild type; YPD, yeast extract–peptone–dextrose.

To test whether *ATG1* and *ATG13* mRNAs are bona fide targets of Dhh1, we first checked their expression when the *DHH1* gene was deleted. The Atg1 and Atg13–PA protein levels were substantially decreased in the *dhh1Δ* cells compared to the WT cells after 6 h of nitrogen starvation (Fig 2A and 2B). Under nutrient-rich conditions, a higher Atg1 protein level was observed in the *dhh1Δ* cells compared to WT cells, consistent with the suggested role of Dhh1 in promoting decapping of some *ATG* mRNAs in this condition [16] (Fig 2A). As controls, we examined *ATG2* and vacuolar protein sorting 30 (*VPS30*)/*ATG6* mRNAs, which did not contain structured regions shortly after start codons (S2A Fig). We also did not observe decreased Atg2–PA and Vps30/Atg6 protein levels in the *dhh1Δ* cells compared to the WT cells after nitrogen starvation (S2B and S2C Fig). By quantitative reverse transcription PCR (RT-qPCR), we found that there were no significant reductions in the *ATG1* and *ATG13* mRNA levels with the deletion of *DHH1* under starvation conditions (Fig 2C). Moreover, when Dhh1 was conditionally knocked down via the AID system, we also observed a lower Atg1 protein level under nitrogen-starvation conditions (Fig 2D). Taken together, these data are consistent with the hypothesis that Dhh1 may promote Atg1 and Atg13 translation during nitrogen starvation. However, Dhh1 is not essential for translation of the two proteins. Accordingly, compared to WT cells, the strain deleted only for *DHH1* showed partial defects in cell viability after 10 d of nitrogen starvation, whereas the *atg1Δ* and the *atg13Δ* cells displayed complete loss of viability after the same treatment (S2D Fig).

### DDX6 regulates ATG16L1 translation

Next, we decided to test whether regulation of Atg protein translation by Dhh1 is conserved in more-complex eukaryotes. To find potential targets of DDX6, the mammalian homolog of Dhh1, we analyzed the structures of some core mammalian autophagy-related mRNAs using SPARCS. Although unc-51 like autophagy activating kinase 1 (*ULK1*; one of the *ATG1* homologs) was not predicted to have structured regions in its transcript, such regions were predicted for *ATG16L1* (S3A Fig). Therefore, we tested whether *ATG16L1* is subjected to DDX6-dependent regulation in the context of amino acid starvation.

In WT HEK293A cells, the ATG16L1 protein level, but not the *ATG16L1* mRNA level, increased significantly after 8 and 24 h of amino acid starvation compared to that under normal conditions, suggesting that *ATG16L1* translation is up-regulated during amino acid starvation (S3B–S3D Fig). In contrast, the ULK1 protein level decreased after amino acid starvation (S3B Fig). In the *DDX6* knockout cells, the basal *ATG16L1* mRNA and ATG16L1 protein levels were higher under normal conditions (S3E Fig). In contrast to the WT, however, upon amino acid starvation the ATG16L1 protein level in the *DDX6* knockout cells did not display a further increase; instead, there was a significant reduction in the ATG16L1 protein level 24 h after amino acid starvation (S3B and S3C Fig), implying that the knockout cells could not up-regulate *ATG16L1* mRNA translation. As a control, the ULK1 protein level was not altered in the *DDX6* knockout cells after amino acid starvation relative to the WT. The results collectively suggest that DDX6 is necessary for translational up-regulation of *ATG16L1* during amino acid starvation in HEK293A cells, indicating that some aspects of translational control are conserved.

### Dhh1 associates with *ATG1* and *ATG13* mRNAs during nitrogen starvation

**Fig 3:**
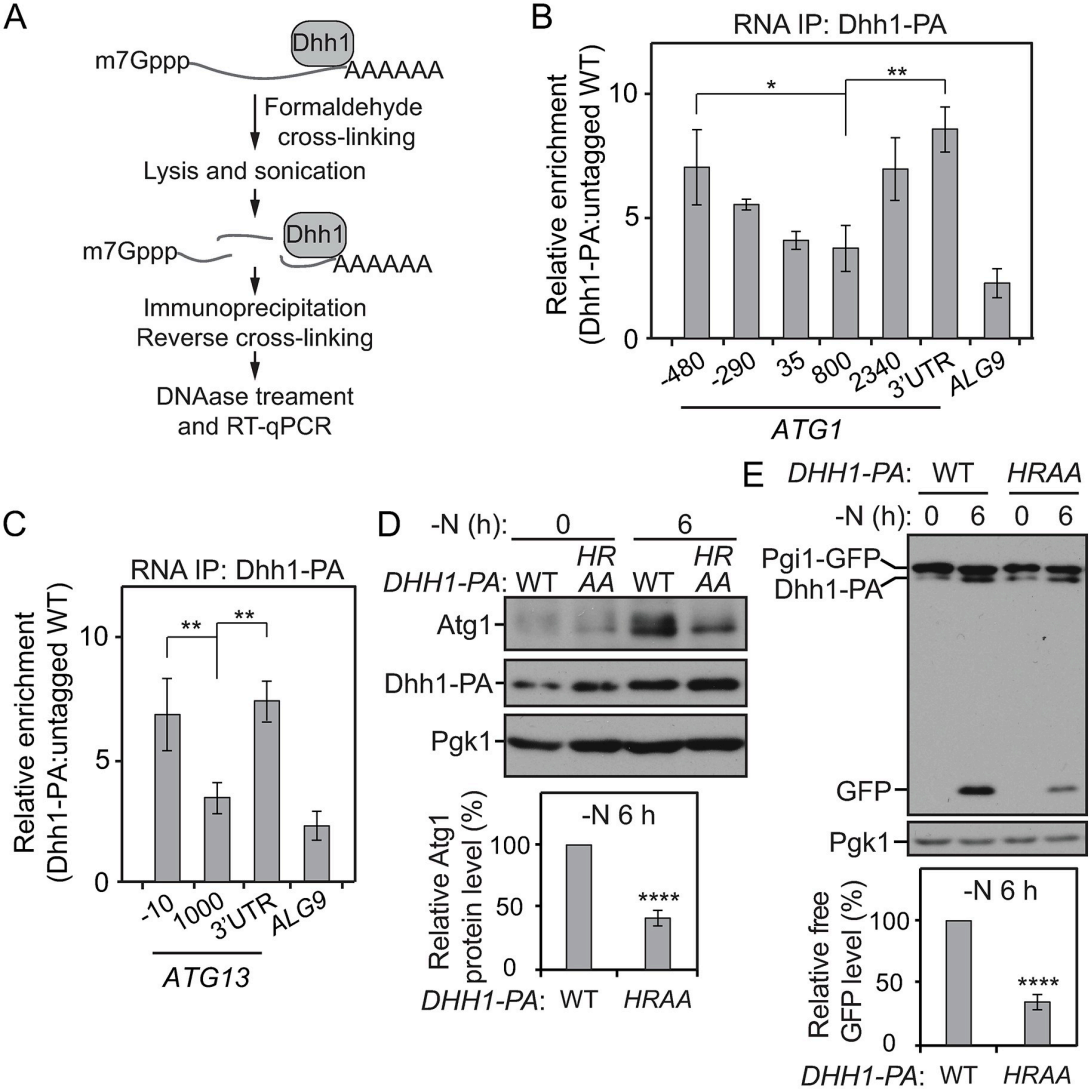
Dhh1 associates with *ATG1* and *ATG13* mRNAs during nitrogen starvation. (A) Workflow of the RNA IP assay. (B, C) WT (SEY6210) and Dhh1–PA (XLY323) cells were grown in YPD to mid-log phase (-N, 0 h) and then shifted to SD-N for 2 h. Cells were subjected to the RNA IP as described in the Materials and methods. RT-qPCR was performed to analyze the enrichment of RNA fragments as indicated. The values were first normalized to the input samples. Next, levels of affinity-isolated RNA fragments from the Dhh1–PA cells were normalized to that from WT cells. *ALG9* mRNA was used as a negative control. Enrichment of regions in *ATG1* and *ATG13* mRNAs is shown in (B) and (C), respectively. *p < 0.05. **p < 0.01. (D, E) Pgi1–GFP Dhh1^WT^–PA (XLY327), and Pgi1–GFP HRAA–PA (XLY328) cells were grown in YPD to mid-log phase (-N, 0 h) and then shifted to SD-N for 6 h. Cell lysates were prepared, subjected to SDS-PAGE, and analyzed by western blot. The analysis of Atg1 protein levels and Pgi1–GFP processing are shown in (D) and (E), respectively. The quantification of Atg1 and free GFP level was conducted as indicated in Figs 2A and 1D, respectively. ****p < 0.0001. (See also S4 Fig; raw numerical values are shown in S1 Data). *ALG9*, asparagine-linked glycosylation 9; Atg, autophagy-related; GFP, green fluorescent protein; HRAA, Dhh1^H395A,R396A^; IP, immunoprecipitation; PA, protein A; Pgi1, phosphoglucoisomerase 1; RT-qPCR, quantitative reverse transcription PCR; SD-N, synthetic minimal medium lacking nitrogen; WT, wild type; YPD, yeast extract–peptone–dextrose.

To further test our hypothesis that Dhh1 regulates Atg1 and Atg13 translation, we examined whether Dhh1 associates with these two mRNAs under nitrogen-starvation conditions. The RNA immunoprecipitation (IP) assay has been utilized to investigate protein–RNA interactions [24]. We immunoprecipitated Dhh1–PA with immunoglobulin G (IgG)-conjugated Sepharose beads from lysates prepared from cells starved for nitrogen (Fig 3A). As a control, nitrogen-starved lysates from cells in which Dhh1 was not tagged were subjected to the same procedures in parallel. Subsequently, the affinity-isolated RNA fragments were analyzed by RT-qPCR. Through this approach, we found that, relative to the control group, significantly more *ATG1* and *ATG13* mRNA fragments were precipitated with Dhh1–PA (Fig 3B and 3C). In addition, the strain expressing Dhh1–PA showed a higher enrichment at both the 5′-UTR and the 3′-UTR regions of *ATG1* and *ATG13* mRNAs. This latter finding suggests that Dhh1 may bind to these transcripts at both of these regions. Moreover, Dhh1–PA showed no or limited binding to most other *ATG* mRNAs (S4A Fig), implying that Dhh1 specifically interacts with *ATG1* and *ATG13* mRNAs under nitrogen-starvation conditions.

To test whether the 5′-UTR and/or the 3′-UTR regions of *ATG1* mRNA is required for regulation by Dhh1, we constructed strains expressing different versions of *ATG1* mRNAs in which either the 5′-UTR or the 3′-UTR region was exchanged. When the native promoter of *ATG1* on the chromosome was switched to the plasma membrane proteolipid 3 (*PMP3*) promoter (which is not predicted to be affected by Dhh1), deletion of *DHH1* did not lead to decreased Atg1 protein levels compared to the control cells (S4B Fig). We also generated *atg1Δ* strains in which *ATG1* was expressed under its native promoter and with either the *ATG1* or *ATG7* 3′-UTR. Depletion of *DHH1* resulted in decreased Atg1 protein levels in the cells expressing the *ATG1* mRNA with its native 3′-UTR (S4C Fig). Conversely, knocking out the *DHH1* gene did not result in less Atg1 translation in the *atg1Δ* cells complemented with the *ATG1* mRNA having the *ATG7* 3′-UTR (S4C Fig). These data suggest that both the 5′-UTR and the 3′-UTR regions of *ATG1* mRNA are very likely to be necessary for Dhh1-dependent translational regulation. Similar results were observed when the native 3′-UTR of *ATG13* mRNA was replaced with the *ATG7* 3′-UTR (S4D Fig), further demonstrating the importance of the 3′-UTR regions in the *ATG* mRNAs in terms of translational regulation by Dhh1.

The residues in Dhh1 that are important for its RNA binding have been identified previously. For example, the Dhh1^H369A^ and Dhh1^R370A^ mutants bind RNAs less efficiently both in vitro and in yeast cells [25]. To further demonstrate that RNA binding of Dhh1 is required for its role in regulating *ATG* translation and autophagy, we generated a yeast strain that stably expresses the Dhh1^H369A,R370A^–PA mutant (“HRAA” in Fig 3D). After nitrogen starvation, the Atg1 protein level in the mutant cells was significantly lower than that in the cells expressing WT Dhh1–PA (Fig 3D). The autophagy activity was also diminished in the Dhh1^H369A,R370A^–PA mutant cells tested by the Pgi1–GFP-processing assay (Fig 3E). Therefore, we conclude that Dhh1 binds to *ATG1* and *ATG13* mRNAs and that the RNA binding is necessary for its role in promoting their translation and autophagy under nitrogen-starvation conditions.

### The structured regions in *ATG1* and *ATG13* ORFs are necessary for the translational regulation by Dhh1 after nitrogen starvation

**Fig 4:**
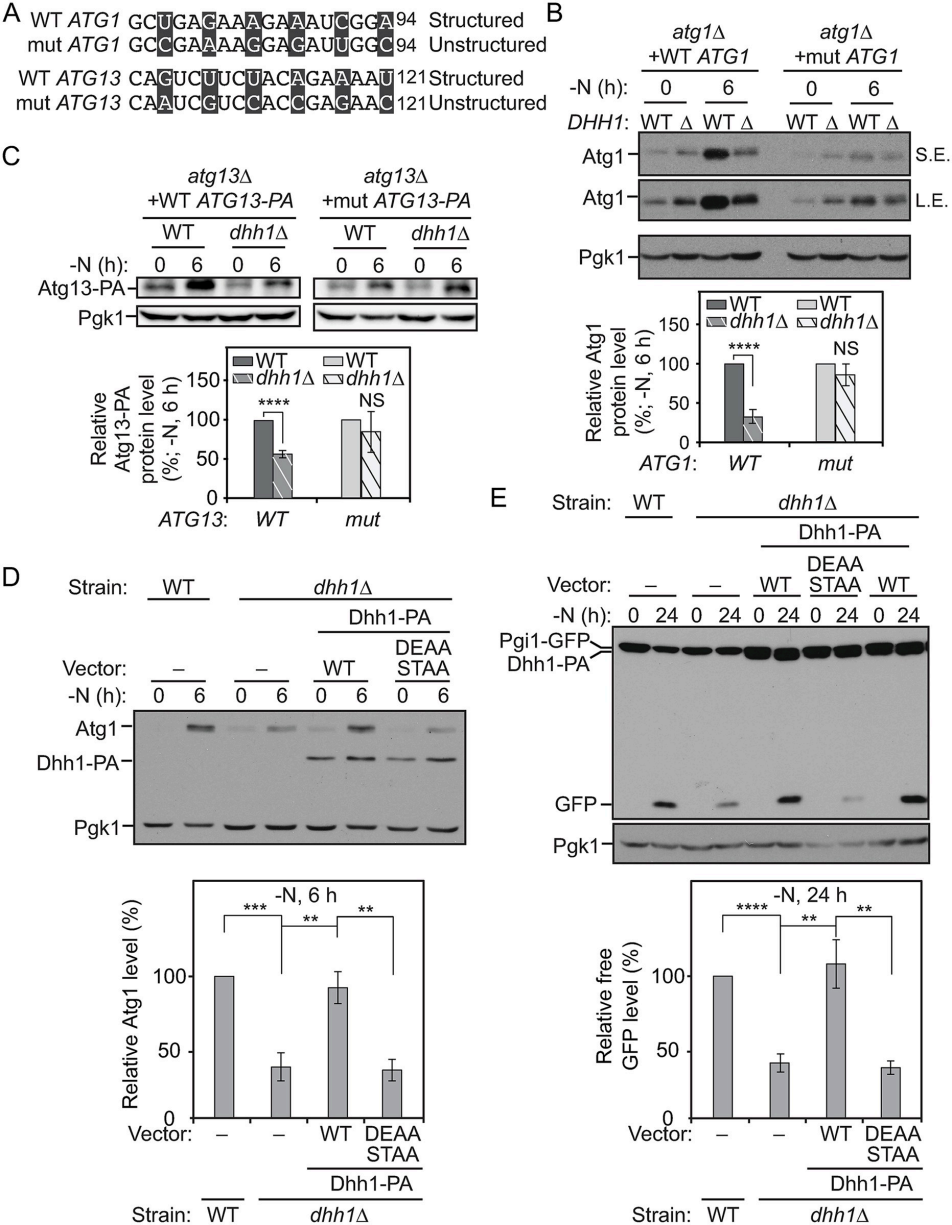
The structured regions in the *ATG1* and *ATG13* ORFs are necessary for the translational regulation by Dhh1 after nitrogen starvation. (A) The mutations (“mut”) made in the structured regions of *ATG1* and *ATG13* ORFs are shown as indicated; mutated nucleotides are presented in black boxes. (B) WT Atg1 (XLY316), WT Atg1 *dhh1Δ* (XLY317), mutant Atg1 (XLY318), and mutant Atg1 *dhh1Δ* (XLY319) cells were grown in YPD to mid-log phase (-N, 0 h) and then shifted to SD-N for 6 h. Cell lysates were prepared, subjected to SDS-PAGE, and analyzed by western blot. The quantification of Atg1 protein level was conducted as indicated in Fig 2A. NS, not significant. ****p < 0.0001. (C) WT Atg13–PA (ZYY202), WT Atg13–PA *dhh1Δ* (ZYY203), mutant Atg13–PA (ZYY205), and mutant Atg13–PA *dhh1Δ* (ZYY206) cells were grown in YPD to mid-log phase (-N, 0 h) and then shifted to SD-N for 6 h. Cell lysates were prepared, subjected to SDS-PAGE, and analyzed by western blot. The quantification of Atg13–PA protein level was conducted as indicated in Fig 2A. The 5′-UTR and 3′-UTR of *ATG13* in these strains were not changed. ****p < 0.0001. (D) The WT strain with empty vector (XLY329), the *dhh1Δ* strain with either empty vector (XLY331), or vectors expressing WT Dhh1–PA (XLY333) or Dhh1^D195A,E196A,S226A,T228A^–PA (DEAA STAA; XLY342) were grown in YPD to mid-log phase (-N, 0 h) and then shifted to SD-N for 6 h. Cell lysates were prepared, subjected to SDS-PAGE, and analyzed by western blot. The quantification of the Atg1 protein level was conducted as indicated in Fig 2A. **p < 0.01. ***p < 0.001. (E) The strains in (D) were grown in YPD to mid-log phase (-N, 0 h) and then shifted to SD-N for 24 h. Cell lysates were prepared and subjected to SDS-PAGE. The processing of Pgi1–GFP was analyzed by western blot. The quantification of free GFP level was conducted as indicated in Fig 1D. **p < 0.01. ****p < 0.0001. (See also S5 Fig; raw numerical values are shown in S1 Data). Atg, autophagy-related; DEAA STAA, Dhh1^D195A,E196A,S226A,T228A^; GFP, green fluorescent protein; L.E., long exposure; NS, not significant; ORF, open reading frame; PA, protein A; Pgi1, phosphoglucoisomerase 1; Pgk1, 3-phosphoglycerate kinase 1; SD-N, synthetic minimal medium lacking nitrogen; S.E., short exposure; WT, wild type; YPD, yeast extract–peptone–dextrose.

Based on the observation that Dhh1 plays a role in the efficient translation of *ATG1* and *ATG13* mRNA, we decided to further investigate whether the predicted structured regions shortly after the start codons of these genes are required for this regulation. We constructed plasmids expressing either WT Atg1 or WT Atg13–PA or their mutant versions by changing the third base of the codons near the structured regions to make them unstructured without modifying the amino acid sequence (Fig 4A and S5A Fig). These plasmids were integrated into the *atg1Δ* or *atg13Δ* cells, respectively. Subsequently, the *DHH1* gene was deleted in these strain backgrounds. In the cells expressing WT Atg1 or WT Atg13–PA, depletion of *DHH1* caused a substantial decrease of the protein levels after nitrogen starvation (Fig 4B and 4C). In contrast, the mutant Atg1 and mutant Atg13–PA total protein levels were lower than seen with the WT controls, but the levels were not significantly affected by knocking out *DHH1* (Fig 4B and 4C). These results suggest that the structured region shortly after the start codons of *ATG1* and *ATG13* mRNAs are necessary for their Dhh1-dependent up-regulated translation after starvation.

As an RNA helicase, Dhh1 was previously hypothesized to modify the structured regions of its target mRNAs so that the translation initiation occurs more efficiently [22]. The “DExD/H” and “SAT” motifs are residues important for the helicase activity of the proteins in this family, and the motifs are highly conserved [26,27]. To test the hypothesis with regard to *ATG1* and *ATG13*, we made constructs stably expressing Dhh1^D195A,E196A^–PA, Dhh1^S226A,T228A^–PA, or Dhh1^D195A,E196A,S226A,T228A^–PA, which are expected to display diminished helicase activity. Compared to the *dhh1Δ* cells expressing WT Dhh1–PA, significantly lower Atg1 protein levels were observed in the *dhh1Δ* cells with Dhh1^D195A,E196A,S226A,T228A^–PA under nitrogen-starvation conditions (Fig 4D). Limited or no defects in Atg1 translation or Pgi1–GFP processing were observed in the cells with either Dhh1^D195A,E196A^–PA or Dhh1^S226A,T228A^–PA (S5B and S5C Fig). It is possible that residual helicase activity of the two mutants was sufficient to drive Atg1 translation as efficiently as the WT protein. Based on the Pgi1–GFP-processing assay, autophagy activity was also impaired in the *dhh1Δ* cells with Dhh1^D195A,E196A,S226A,T228A^–PA, compared to that in cells with WT Dhh1–PA (Fig 4E). These observations collectively suggest that efficient translation of *ATG1* and *ATG13* mRNAs is dependent on their structured regions in the ORFs and the helicase activity of Dhh1 under nitrogen-starvation conditions.

### Dhh1 interacts with Eap1 under nitrogen-starvation conditions

Dhh1 has recently been shown to associate with the translation initiation machinery [22], but it is not known whether Dhh1 directly binds to the translation initiation complex, nor is it clear how the association is regulated after nitrogen starvation. In eukaryotic cells, translation occurs mostly in a cap-dependent manner, which requires the formation of the EIF4F complex, including EIF4A, EIF4E, and EIF4G on the 5′-UTR of mRNAs. EIF4G is a scaffold for the complex and interacts with EIF4E, which directly binds to the 5′ mRNA cap [28]. When mechanistic target of rapamycin kinase complex 1 (MTORC1) kinase activity is inhibited upon nitrogen starvation, EIF4EBPs inhibit translation through competing with EIF4G to bind EIF4E [28].

Although EIF4EBPs generally inhibit translation, they play positive roles in the translation of some mRNAs in breast cancer cells under hypoxic conditions [29]. In addition, *Thor/4E-BP1* null flies are more sensitive to starvation compared to WT flies [30]. These studies indicate that EIF4EBPs may function positively on certain mRNAs under specific stress conditions. In yeast, Eap1, an EIF4EBP, physically interacts with Dhh1 when nutrients are rich [31], leading us to propose that Eap1 plays a role in regulating *ATG* mRNA translation by modulating the interaction between Dhh1 and the translation initiation machinery. In addition, Eap1 is not essential and targets only a subset of mRNAs, making it a good candidate for further analysis.

**Fig 5:**
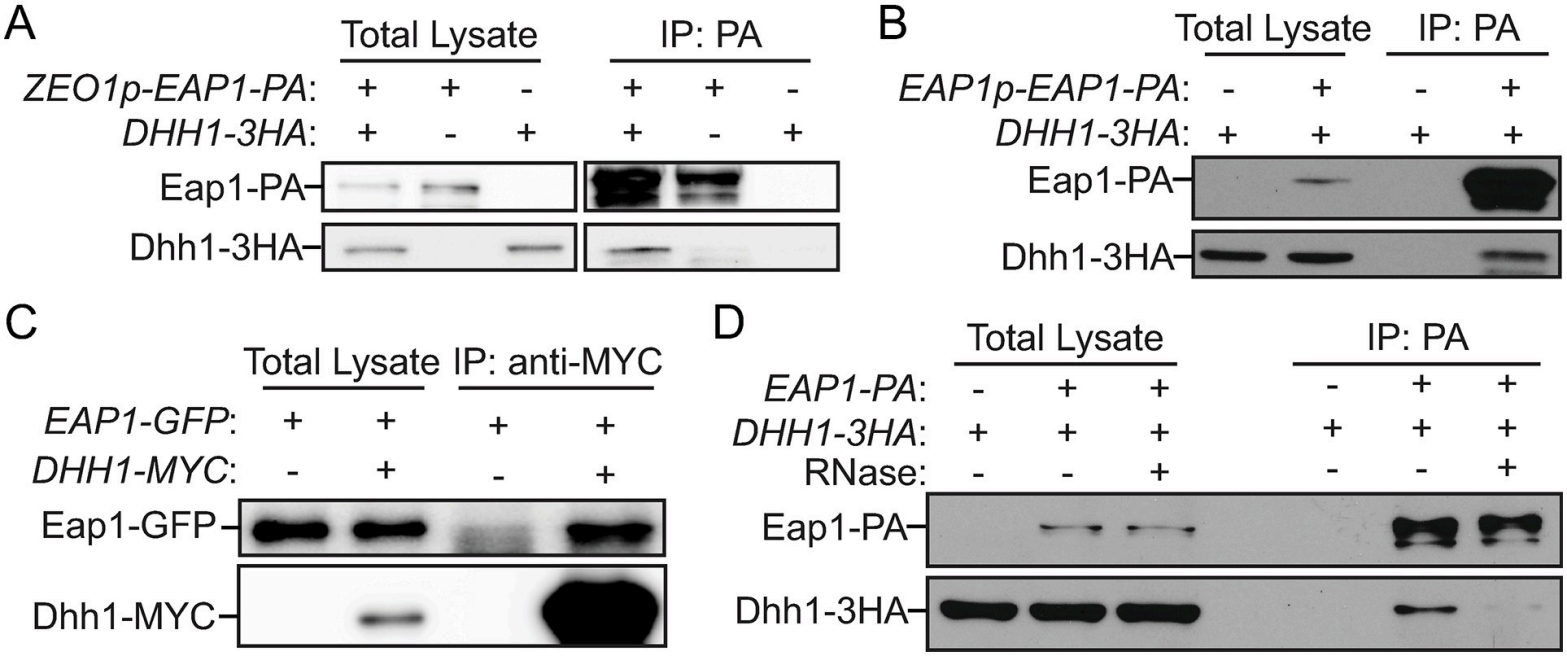
Dhh1 interacts with Eap1 under nitrogen-starvation conditions. (A) *ZEO1p–EAP1–PA* (ZYY207; *ZEO1p*), *DHH1–3HA* (ZYY208), and *ZEO1p–EAP1–PA DHH1–3HA* (ZYY209) cells were grown in YPD to mid-log phase (-N, 0 h) and then shifted to SD-N for 2 h. The samples were collected and subjected to the protein–protein IP procedures described in the Materials and methods. The analysis of the samples by western blot is shown. (B-D) Cells were cultured and subjected to procedures as indicated in (A). The analysis of the samples by western blot is shown: (B) *DHH1*–*3HA* (ZYY208) and *DHH1*–*3HA EAP1p*–*EAP1*–*PA* (XLY345; *EAP1* promoter); (C) *ZEO1p*–*EAP1*–*GFP* (XLY346) and *DHH1*–*MYC ZEO1p*–*EAP1*–*GFP* (YZY256); (D) *DHH1*–*3HA* (ZYY208) and *ZEO1p*–*EAP1*–*PA DHH1*–*3HA* (ZYY209). The RNase treatment during incubation with IgG beads was conducted as described in the Materials and methods. (See also S6 Fig). Eap1, eukaryotic translation initiation factor 4E–associated protein 1; GFP, green fluorescent protein; IgG, immunoglobulin G; IP, immunoprecipitation; *PA*, protein A; SD-N, synthetic minimal medium lacking nitrogen; YPD, yeast extract–peptone–dextrose; *ZEO1p*, zeocin resistance 1 promoter.

First, we examined whether Eap1 interacts with Dhh1 under nitrogen-starvation conditions. We immunoprecipitated either endogenous or overexpressed levels of Eap1–PA with IgG-conjugated Sepharose beads from lysates of cells subjected to nitrogen starvation. The Dhh1–3HA protein was affinity-isolated together with Eap1–PA in both cases (Fig 5A and 5B, and S6A Fig). In addition, when Dhh1–MYC from yeast cell lysates was immunoprecipitated with anti-MYC beads, a significant amount of Eap1–GFP protein was also precipitated (Fig 5C). Finally, when the samples were treated with RNase, the amount of Dhh1–3HA affinity isolated by Eap1–PA significantly decreased (Fig 5D). These results collectively demonstrate that Dhh1 interacts with Eap1 in an RNA-dependent manner under nitrogen-starvation conditions.

To further characterize the interaction between Dhh1 and Eap1, we decided to map the regions of the two proteins mediating the interaction. We started the analysis with predictions of intrinsically disordered regions (IDRs) by IUPred2 and disordered binding regions by ANCHOR2 in Dhh1 and Eap1 [32]. The C terminus of Dhh1 contains a disordered binding region (amino acids 440–506) that may mediate dynamic protein–protein interactions (S6B Fig). The Eap1 protein is highly disordered and has several disordered binding regions; only residues 80–150 in Eap1 are predicted to be ordered and/or structured (S6C Fig).

Based on the information from the predictions, we constructed yeast strains expressing Eap1–PA, together with either WT Dhh1–3HA or Dhh1[1–425]–3HA, which lacks the predicted disordered binding region. When Eap1–PA was immunoprecipitated with IgG-coated beads, WT Dhh1–3HA, but not Dhh1[1–425]–3HA, was pulled down, suggesting that the C-terminal 81 amino acids of Dhh1 are required for the interaction (S6D Fig). We also determined that residues 1–270 of Eap1 are sufficient to bind to WT Dhh1–3HA (S6E Fig).

### Eap1 facilitates Atg1 and Atg13 translation during nitrogen starvation

**Fig 6:**
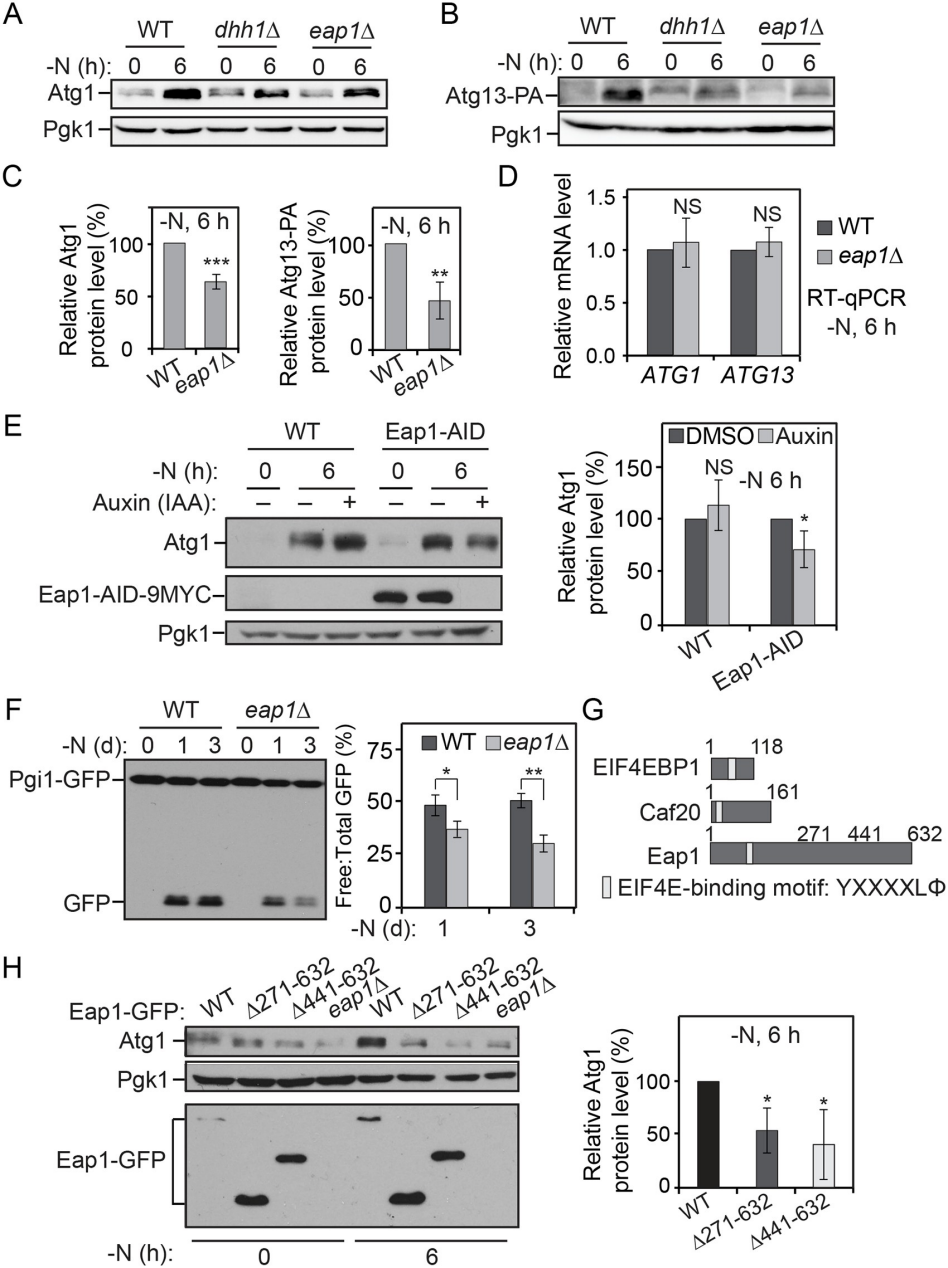
Eap1 facilitates Atg1 and Atg13 translation during nitrogen starvation. (A, B, C) Atg13–PA (ZYY202) and Atg13–PA *eap1Δ* (ZYY204) cells were grown in YPD to mid-log phase (-N, 0 h) and then shifted to SD-N for 6 h. Cell lysates were prepared, subjected to SDS-PAGE, and analyzed by western blot. Atg1 and Atg13–PA levels are shown in (A) and (B), respectively. The quantification of Atg1 and Atg13–PA protein levels was conducted as indicated in Fig 2A and was shown in (C). **p < 0.01. ***p < 0.001. (D) Atg13–PA (ZYY202) and Atg13–PA *eap1Δ* (ZYY204) cells were grown in YPD to mid-log phase (-N, 0 h) and then shifted to SD-N for 6 h. Total RNA for each sample was extracted, and the *ATG1* and *ATG13* mRNA levels were quantified by RT-qPCR. (E) WT (XLY338) and Eap1–AID (ZYY210) cells were grown in YPD to mid-log phase and treated with either DMSO or 300 μM IAA for 30 min. They were then shifted to SD-N for 6 h in the presence of either DMSO or IAA. Cell lysates were prepared, subjected to SDS-PAGE and analyzed by western blot. The quantification of Atg1 levels was conducted as indicated in Fig 2D. *p < 0.05. (F) Pgi1–GFP (XLY306) and Pgi1–GFP *eap1Δ* (XLY310) cells were grown in YPD to mid-log phase (-N, 0 d) and then shifted to SD-N for 1 or 3 d. Cell lysates were prepared, subjected to SDS-PAGE, and analyzed by western blot. The quantification of the ratio of free to total GFP was conducted as indicated in Fig 1C. *p < 0.05. **p < 0.01. (G) Schematic images of the domains of EIF4EBPs, including EIF4EBP1 (*Homo sapiens*), Caf20 (*Saccharomyces cerevisiae*), and Eap1 (*S*. *cerevisiae*). Although they share an EIF4E binding motif, Eap1 has a long C-terminal stretch that is absent in the other two proteins. (H) WT Eap1–GFP (ZYY215), Eap1^Δ271–632^–GFP (ZYY211), Eap1^Δ441–632^–GFP (ZYY212), and *eap1Δ* (ZYY204) cells were grown in YPD to mid-log phase (-N, 0 d) and then shifted to SD-N for 6 h. Cell lysates were prepared, subjected to SDS-PAGE, and analyzed by western blot. The quantification of Atg1 protein levels was conducted as indicated in Fig 2A. *p < 0.05. (See also S7 Fig; raw numerical values are shown in S1 Data). AID, auxin-inducible degron; Atg, autophagy-related; Caf20, cap associated factor 20; DMSO, dimethyl sulfoxide; Eap1, EIF4E–associated protein 1; EIF4E, eukaryotic translation initiation factor 4E; EIF4EBP, EIF4E binding protein; GFP, green fluorescent protein; IAA, indole-3-acetic acid; NS, not significant; PA, protein A; Pgi1, phosphoglucoisomerase 1; Pgk1, 3-phosphoglycerate kinase 1; RT-qPCR, quantitative reverse transcription PCR; SD-N, synthetic minimal medium lacking nitrogen; WT, wild type; YPD, yeast extract–peptone–dextrose.

To test the roles of Eap1 in Atg protein translation and autophagy, we deleted the *EAP1* gene and checked its effects on *ATG1* and *ATG13* expression and autophagy activity. We observed lower Atg1 and Atg13–PA protein levels in the *eap1Δ* cells compared to the WT cells (Fig 6A–6C), even though the *ATG1* and *ATG13* mRNA levels were not significantly affected (Fig 6D). Conditional knock-down of Eap1 with the AID system also led to a decreased Atg1 protein level under starvation conditions (Fig 6E). In addition, autophagy activity was compromised in the *eap1Δ* cells based on the Pgi1–GFP-processing assay (Fig 6F). These results suggest that Eap1 promotes Atg1 and Atg13 translation and autophagy under nutrient-depletion conditions.

In yeast cells aside from Eap1, another EIF4EBP, cap associated factor 20 (Caf20), was reported previously [33,34]. Deletion of *CAF20* did not cause decreased Atg1 translation, nor did it affect the autophagy activity under nitrogen-starvation conditions (S7 Fig). When the protein sequences of Caf20, Eap1, and the mammalian EIF4EBP1 were compared, we found that although they share a conserved EIF4E interaction motif in the N terminus, Eap1 uniquely contains a long C-terminal region (Fig 6G). Although this region is not necessary for its interaction with Dhh1 (S6E Fig), we decided to test whether this region is important for facilitating Atg protein translation. In cells stably expressing the Eap1[Δ271–632]–GFP and Eap1[Δ441–632]–GFP mutants where the corresponding part of the C-terminal region was truncated, we observed lower Atg1 protein levels compared to cells with WT Eap1–GFP (Fig 6H). These results suggest that the long C-terminal region of Eap1, which is absent in other EIF4EBPs, is required for its role in promoting Atg1 translation under nitrogen-starvation conditions.

## Discussion

**Fig 7:**
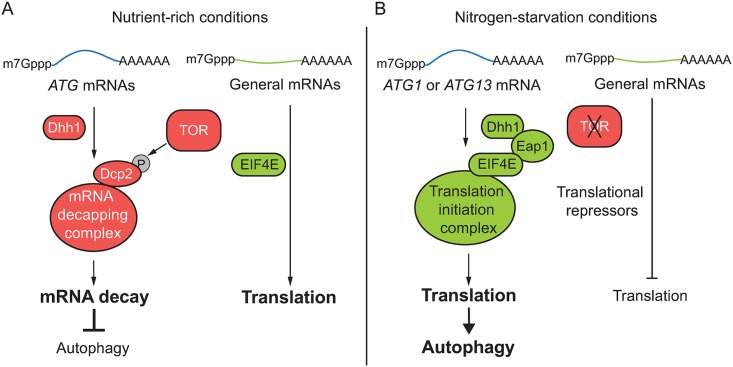
A model for the dual roles of Dhh1 in regulating autophagy under different conditions. (A) Under nutrient-rich conditions, Dhh1 delivers some *ATG* mRNAs such as *ATG8* transcripts to the mRNA decapping machinery to mediate their degradation, maintaining *ATG* mRNAs and autophagy at basal levels [16]. TOR is active under this condition and phosphorylates Dcp2, the major decapping enzyme, to promote its decapping activity [16]. However, general mRNAs are actively translated when TOR is active. (B) Upon nitrogen starvation, TOR is inhibited and the translation of general mRNAs is repressed. In contrast, Dhh1 associates with *ATG1* and *ATG13* mRNAs and interacts with Eap1, an EIF4E binding protein, facilitating their delivery to the translation initiation complex to promote their translation and thus autophagy activity. *ATG*, autophagy-related; Dcp2, mRNA decapping 2; Eap1, EIF4E–associated protein 1; EIF4E, eukaryotic translation initiation factor 4E; TOR, target of rapamycin.

The DExD/H-box RNA helicases Dhh1 and its Vad1 homolog in *Cryptococcus neoformans* bind certain *ATG* mRNAs and direct them to the decapping enzyme Dcp2 for degradation under nutrient-rich conditions, maintaining autophagy at a basal level [16]. When autophagy is induced by nutrient deprivation, inhibition of TOR leads to the loss of Dcp2 phosphorylation and a subsequent decrease in decapping activity. However, Vad1 or Dhh1 still show binding affinity toward some *ATG* mRNAs after nutrient deprivation, though to a lesser extent than that seen in nutrient-rich conditions [16]. Because the *ATG* mRNA levels in the *dhh1Δ* cells under starvation conditions are similar to those in WT cells, the binding of Dhh1 to the *ATG* mRNAs seen in these conditions may not be related to mRNA stability. Here, we found that Dhh1 promotes autophagy by targeting *ATG1* and *ATG13* mRNAs to the translation initiation machinery after nutrient deprivation, revealing the bidirectional roles of Dhh1 in regulating autophagy (Fig 7). These observations also suggest that Dhh1 is able to deliver mRNAs to different RNA processing machineries, resulting in distinct fates of the mRNAs—under growing conditions, Dhh1 targets certain *ATG* mRNAs for degradation but subsequently acts as a factor that promotes autophagy by facilitating *ATG* mRNA translation during starvation.

What are the factors determining the outcomes of these mRNAs? Our analysis implicates specific elements within the mRNAs. When the structured regions shortly after the start codons of *ATG1* and *ATG13* mRNAs were mutated to be unstructured or disordered, translation of the mRNAs was no longer promoted by Dhh1 after starvation. The regions that Dhh1 binds may also contribute to determining their fates. We showed that Dhh1 associates with both the 5′-UTR and the 3′-UTR of *ATG1* mRNA. When either the native 5′-UTR or the 3′-UTR of *ATG1* mRNA was replaced by the corresponding elements from genes not affected by Dhh1, the mutant mRNAs were not translationally regulated by Dhh1. In mammalian cells, it was reported that DDX6 binds to the 3′-UTR in the mRNAs of positive regulators of proliferation and/or self-renewal such as cyclin dependent kinase 1 (*CDK1*) and enhancer of zeste 2 polycomb repressive complex 2 subunit (*EZH2*) to facilitate their translation in epidermal progenitor cells [35]. In contrast, DDX6 binds to the 5′-UTR of Kruppel like factor 4 (*KLF4*), which encodes a differentiation-inducing transcription factor to mediate its degradation through the decapping pathway. Therefore, some intrinsic feature(s) inside the regions Dhh1 binds may also be critical for deciding what to do with the bound mRNAs. Conversely, regulation of interactions between Dhh1 and the different RNA function machineries may also be indispensable, though this is largely unexplored. Is it possible that posttranslational modifications of Dhh1 are involved? Does the binding to mRNAs induce different conformational changes of Dhh1 that favor distinct protein–protein interactions?

Cap-dependent translation initiation requires formation of an EIF4F complex on mRNAs. The EIF4F complex is composed of three major components: EIF4A, EIF4E, and EIF4G [36]. EIF4EBPs inhibit translation initiation by competing with EIF4G to bind EIF4E [37]. TOR, an autophagy inhibitor, positively regulates cap-dependent translation by allowing the replacement of EIF4EBPs with EIF4G under nutrient-rich conditions [28]. Upon nutrient deprivation, TOR is inhibited, resulting in translational repression of approximately 98% of the cellular genes. This is partly due to dephosphorylation and activation of EIF4EBPs upon TOR inhibition, which in turn suppresses complex formation between EIF4G and EIF4E [28,38]. However, Eap1, a yeast EIF4EBP, unexpectedly promotes *ATG1* and *ATG13* mRNA translation under starvation conditions. This function is specific to Eap1 in that the other known EIF4EBP in yeast, Caf20, does not play a role in regulating the translation of these two mRNAs or autophagy activity. Eap1 has a unique long C-terminal region that is absent in both Caf20 and its mammalian homologs. This region may distinguish Eap1 as a positive regulator of *ATG1* and *ATG13* mRNAs. It would be interesting to identify a functional counterpart of the C-terminal part of Eap1 in higher eukaryotes; it is possible that the domain’s function is executed by some uncharacterized protein(s) in mammals.

Eap1 was reported to physically interact with Dhh1, coordinating with the decapping complex to degrade translationally suppressed mRNAs under nutrient-rich conditions [31]. However, the Eap1–Dhh1 complex facilitates the translation of *ATG1* and *ATG13* mRNAs upon nutrient deprivation. Nonetheless, the signaling that controls the switch of roles is unknown. The long C-terminal region of Eap1 is not required for its interaction with Dhh1, but it is necessary for promoting Atg protein translation. This region is highly phosphorylated [39–42]. It is very likely that the phosphorylation regulation of Eap1 by TOR and/or other kinases and the potential interaction partners of Eap1 through its C-terminal region are important in regulating the switch of the roles of the Eap1–Dhh1 complex.

We propose that Dhh1 helicase activity is required for its function based on sequence homology and the effect of the D195A E196A S226A T228A quadruple mutant (Fig 4). However, we have not been able to verify the effect of these mutations on helicase activity in vitro. A previous study demonstrated that WT Dhh1 does not display in vitro helicase activity [43]. This apparent lack of activity is likely due to a requirement for accessory factors, as demonstrated for the EIF4A DEAD-box helicase [26], and Dhh1 helicase accessory factors have not yet been identified. Furthermore, the DEAD-box motif mutant is likely defective in ATPase activity [27,44], which is a prerequisite for helicase activity. Based on the crystal structure of Dhh1 [25], however, the DEAD-box motif is located away from residues shown to be required for RNA binding. Thus, although we cannot rule out the possibility that the quadruple mutant generated in this study affects Atg1 and Atg13 translation because of defects in binding the corresponding transcripts, we consider this possibility unlikely.

In summary, our study in yeast shows how under conditions of nitrogen starvation, *ATG1* and *ATG13* mRNA translation, which is crucial for maintaining autophagy activity and cell viability, is promoted when general translation is inhibited through a special translation regulatory machinery including structured mRNA, Dhh1, and Eap1 (Fig 7). We also demonstrated that in HEK293A cells under conditions of amino acid starvation, DDX6 facilitates the translation of *ATG16L1* mRNA, which also shows potential structured regions shortly after the start codon. This finding suggests that the mechanism of translational regulation of *ATG* genes involving Dhh1 is conserved in more-complex eukaryotes, although the specific genes that are regulated may differ. During preparation of our manuscript, a study showed that EIF5A promotes *ATG3* translation and autophagy in mammalian cells [45], further demonstrating that specialized molecular mechanisms that underlie the translational up-regulation of autophagy-related genes in stress conditions are critical.

## Materials and methods

### Yeast strains, media, and growth conditions

Yeast strains used in this study are listed in S1 Table. The XLY328 (*DHH1*^*H369A*,*R370A*^*–PA*) strain was made by mutating the *DHH1* gene at the corresponding residues on the genome [46]. For nutrient-rich conditions, yeast cells were grown in YPD medium (1% yeast extract, 2% peptone, 2% glucose). For nitrogen starvation, cells were cultured in SD-N medium (0.17% yeast nitrogen base without ammonium sulfate or amino acids, 2% glucose).

### Generation of *DDX6* knockout cells using the CRISPR-Cas9 system

To generate a *DDX6* knockout in HEK293A cells, the following oligonucleotides encoding the guide RNA was cloned into the pLentiCRISPRv2 plasmid (Addgene, 52961; deposited by Dr. Feng Zhang), which can express both guide RNA and CAS9:

- hDDX6sg1 F, caccgCTATTAAACCTGGTGATGAC
- hDDX6sg1 R, aacGTCATCACCAGGTTTAATAGc

HEK293A cells were transfected with 2 μg of the construct using polyethylenimine and were selected with puromycin for 2 d to remove untransfected cells. Knockout cells were confirmed through western blotting.

### Cell culture and amino acid starvation

HEK293A (Invitrogen, R70507) cells were cultured in Dulbecco’s modified Eagle’s medium (DMEM; Thermo Fisher Scientific, 11965–092) supplemented with 10% fetal bovine serum (FBS; Sigma, F4135) and 1% penicillin-streptomycin (Thermo Fisher Scientific, 15140–122) at 37 °C and 5% CO_2_.

For amino acid starvation, cells were cultured for 48 h in DMEM with 10% FBS, washed twice with PBS, and incubated with amino acid–free RPMI medium (US Biological, R8999-04A) for the indicated times.

### Plasmids

The pRS–ATG1(406) plasmid was made by two-step cloning. The DNA fragments containing either 1,000 base pairs (bps) upstream of the start codon together with the ORF of the *ATG1* gene or 1,000 bps downstream of the *ATG1* ORF were amplified by PCR from WT yeast genomic DNA. The fragments were then ligated into the KpnI and XhoI sites and the XhoI and XmaI sites of the pRS406 vector, resulting in the pRS–ATG1(406) plasmid, based on which the pRS–ATG1^mut^(406) construct was made by site-directed mutagenesis. The plasmid pRS–ATG1–ATG7[3′-UTR](406) was made based on pRS–ATG1(406) via fast cloning as described previously [47]. The fragment containing 1,000 bps downstream of the *ATG7* ORF was amplified by PCR from WT yeast genomic DNA. The native *ATG1* 3′-UTR in the pRS–ATG1(406) plasmid was then replaced by the *ATG7* 3′-UTR using fast cloning.

The pRS–ATG13–PA(406) plasmid was made by fast cloning [47]. The DNA fragment containing 600 bps upstream of the start codon of *ATG13*, the ORF encoding *ATG13–PA*, and 423 bps downstream of the *ATG13* ORF was amplified by PCR from the genomic DNA of a yeast strain in which the *ATG13* gene was C-terminal-tagged with one copy of PA followed by the *ATG13* endogenous 3′-UTR. The fragment was inserted into the pRS406 vector by fast cloning [47]. The insertion takes place between EcoRI and BamHI sites. The pRS–ATG13^mut^–PA(406) plasmid was made by site-directed mutagenesis based on the pRS–ATG13–PA(406) plasmid. The plasmid pRS–ATG13–PA–ATG7[3′-UTR](406) was constructed by replacing the native *ATG13* 3′-UTR in the pRS–ATG13–PA(406) plasmid with the *ATG7* 3′-UTR (1,000 bps downstream of the *ATG7* ORF) via fast cloning.

The pRS–DHH1–PA(405) plasmid was constructed by inserting a fragment containing the 675 bps upstream of the *DHH1* ORF, the *DHH1* ORF, the sequence encoding two tandem repeats of PA, and the *ADH1* terminator into the XhoI and SpeI sites of pRS405. This fragment was amplified by PCR from the genomic DNA of a yeast strain in which the *DHH1* gene was tagged with two tandem repeats of PA followed by the *ADH1* terminator. The pRS–DHH1^D195A,E196A^–PA(405), pRS–DHH1^S226A,T228A^–*PA*(405), and pRS–DHH1^D195A,E196A,S226A,T228A^–PA(405) plasmids were made by site-directed mutagenesis based on pRS–DHH1–PA(405).

### AID system

To set up the AID system, SEY6210 (WT) cells were first transformed with the plasmid pNHK53 (*ADH1p*–*OsTIR1*–*9MYC*). Dhh1 and Eap1 were then tagged with AID–9MYC by homologous recombination. The DNA fragments used for transformation were amplified with either the pKAN–AID*–9MYC or pHIS3–AID*–9MYC (Addgene, 99522 and 99524; deposited by Dr. Helle Ulrich) as the template DNA. The “AID*” refers to the 71–116 amino acids of the IAA17 protein in plants [21].

To conditionally knock down the genes of interest, the cells were first treated with 300 μM IAA (Sigma, I2886) during mid-log phase in YPD medium for 30 min to induce degradation of targeted proteins before they were washed and shifted to SD-N for starvation. IAA (300 μM) was added to SD-N medium to maintain degradation of the chimeric protein. After an appropriate time period of starvation and treatment, samples were collected for western blot analysis.

### RNA IP assay

The RNA IP assay was adapted from the protocol described previously [24]. Dhh1–PA and Dhh1 untagged cells were cultured to mid-log phase in YPD and then were starved for 2 h in SD-N medium. The starved cells were subjected to cross-linking with 0.8% formaldehyde for 10 min before being harvested. The cross-linking was stopped by the addition of glycine. After washing with PBS, the samples were resuspended in FA lysis buffer (50 mM HEPES [pH 7.5], 150 mM NaCl, 1 mM EDTA, 1% Triton X-100, 0.1% sodium deoxycholate, 0.1% SDS). Before vortexing with glass beads to lyse the cells, PMSF (5 mM final concentration), 1 tablet of protease inhibitor cocktail (Roche), and RNasin PLUS RNase inhibitor (Promega) were added into the buffer. The lysates were collected and sonicated at 4 °C with 3 rounds of 15-s pulses with 45% amplitude. After centrifugation at 10,000*g* for 5 min, the supernatants were recovered. An aliquot of each sample was stored at −80 °C as inputs for later use. The remainder of the supernatant fractions were incubated with IgG Sepharose beads (GE Healthcare Life Sciences) overnight at 4 °C. IP fractions were washed with FA lysis buffer several times and one time with TE buffer (10 mM Tris-HCl [pH 7.5], 1 mM EDTA). Then, the proteins and RNAs were eluted in RIP elution buffer (50 mM Tris-HCl [pH 7.5], 10 mM EDTA, 1% SDS) with RNase inhibitor at 70 °C for 10 min. The IP elution and input samples were reverse cross-linked by incubation with proteinase K for 1 h at 42 °C, followed by 1 h at 65 °C. The RNA in these samples was then recovered with acid-phenol:chloroform (pH 4.5) and subsequent ethanol precipitation. Glycogen (20 μg) was added to the samples to facilitate RNA precipitation. The pellets were washed with 70% ethanol and dried. The samples were next treated using the TURBO DNA-free kit (Thermo Fisher Scientific) to remove residual DNA. The RNA samples were then subjected to analysis by RT-qPCR.

### RT-qPCR

For RT-qPCR in yeast cells, the cells were cultured in YPD medium to mid-log phase. An aliquot was collected as the nutrient-rich sample. The other aliquot was shifted to SD-N medium for 6 h before the cells were collected. The total RNA for each sample was isolated using the RNeasy mini kit (Qiagen). One microgram of total RNA was subjected to reverse transcription using the High Capacity cDNA Reverse Transcription Kit (Applied Biosystems). To analyze the cDNA levels, real-time PCR was performed with Power SYBR Green master mix (Applied Biosystems). The gene-specific primers for *ATG1*, *ATG2*, *ATG13*, and the reference gene *TAF10* are listed in a previous study [16]. The primers used for the RT-qPCR analysis for the RNA IP assays in yeast cells are listed in the supporting information.

For RT-qPCR in HEK293A cells, RNA samples were extracted using Trizol according to the manufacturer’s instructions (Thermo Fisher Scientific, 15596018). RNA (1 μg) was treated with DNase I (Thermo Fisher Scientific, 18068015) and then subjected to reverse transcription using MMLV reverse transcriptase (Thermo Fisher Scientific, 28025013) and random hexamers (Thermo Fisher Scientific, N8080127). The cDNA products were diluted 5-fold with nuclease-free water, and 1 μl of the diluted cDNA was used for real-time qPCR with iQ SYBR Green Supermix (Bio-Rad, 1708884). qPCR was performed using the StepOnePlus Real-Time PCR system (Applied Biosystems). The gene-specific primers used for the RT-qPCR analysis are listed in the supporting information.

### Protein–protein coimmunoprecipitation assay

Yeast cells were cultured in YPD medium to mid-log phase. The cells were washed with sterile water one time before they were shifted to SD-N medium for 2-h starvation. Cells (50 OD units) for each sample were harvested and washed once with ice-cold PBS. Cells were lysed by vortexing them with glass beads in lysis buffer (10×PBS, 200 mM sorbitol, 1 mM MgCl_2_, 1% Tween 20, 1 mM DTT, 1 mM PMSF, protease inhibitor cocktail [Roche Diagnostic, 1 tablet for 500 μl solution], RNasin PLUS RNase inhibitor [Promega]) at 4 °C. After spinning down the cell debris, 50 μl of the supernatant of each sample was saved as total lysate, and 500 μl of the supernatant was subjected to IP. For total lysate, proteins were precipitated by adding 1 ml 10% TCA, and the samples were kept on ice for 30 min before centrifugation. The pellets were washed with acetone once and then air dried. Next, 50 μl MURB (50 mM NaH_2_PO_4_ [pH 7.0], 2.5 mM MES [pH 7.0], 1% SDS, 3 M urea, 0.5% β-mercaptoethanol, 1 mM NaN_3_, 0.2 μg/μl bromophenol blue) was added before the samples were heated at 55 °C for 15 min. For IP, 40 μl IgG Sepharose beads (GE Healthcare Life Sciences) or anti-MYC magnetic beads (Thermo Scientific) were prepared for each sample by washing them with IP buffer (10×PBS, 200 mM sorbitol, 1 mM MgCl_2_, 1% Tween 20) 3 times. Supernatant (500 μl) was added to the prepared beads. The total volume of each sample was adjusted to 1 ml with IP buffer. The samples were incubated at 4 °C on a shaker for 2 to 10 h. The IgG beads were then collected by centrifugation and washed 6 times with IP buffer. After the washing, 50 μl of MURB was added to the samples before they were heated at 55 °C for 20 min. The input and IP samples were then analyzed by western blot.

For RNase treatment, the corresponding samples were suspended in the lysis buffer containing 0.1 μg/μl RNase (Invitrogen), without RNase inhibitor. The samples were incubated at 4 °C on a shaker for 8 to 10 h.

### Western blot

The western blot detecting yeast proteins was performed as described previously [8,17]. The antisera to Pgk1 was generously provided by Dr. Jeremy Thorner (University of California, Berkeley) and used as described previously [8]. Antibody to YFP (Clontech, 632381) was used to detect GFP-tagged proteins. OsTir1–9MYC, Dhh1–AID–9MYC, and Eap1–AID–9MYC were detected by the antibody to the MYC epitope (Sigma, M4439). The antibody to PA (Jackson ImmunoResearch, 323-005-024) was used to detect Atg13–PA, Dhh1–PA, and Eap1–PA. Antibody to the HA epitope (Sigma, H3663) was used to detect Dhh1–3HA. The endogenous antibody to Atg1 was described previously [48].

For western blot detecting proteins in HEK293A cells, whole-cell lysates were prepared in cell lysis buffer (20 mM Tris [pH 7.6], 150 mM NaCl, 1 mM EDTA, 1 mM EGTA, 2.5 mM sodium phosphate, 1 mM glycerol 2-phosphate, 1 mM NaF, 1 mM NaVO_4_, 0.3% CHAPS, 1× EDTA-free protease inhibitor cocktail [Roche, 05 892 791 001]). The lysates were centrifuged at 4 °C for 10 min at 15,000 rpm, and the supernatants were collected. After normalization of protein concentration with the Bradford assay, protein samples were boiled in SDS sample buffer for 5 min, followed by SDS-PAGE, and were transferred to PVDF membranes. The membranes were blocked with 5% skim milk, probed with primary antibodies (1:200, DDX6 [Santa Cruz Biotechnology, sc-376433]; 1:200, ATG16L1 [Santa Cruz Biotechnology, sc-393274]; 1:1,000, ULK1 [Cell Signaling Technology, 4773S]; 1:1,000, ACTB [DSHB, JLA20]), and then probed with horseradish peroxidase–coupled secondary antibodies (1:2,000, anti-rabbit [1706515] and anti-mouse [1706516] antibodies were from Bio-Rad). Chemiluminescence images were obtained using the LAS4000 (GE) system.

### SPARCS

The structured region of mRNAs are predicted using the SPARCS program downloaded from http://csb.cs.mcgill.ca/sparcs/ with ViennaRNA package 2.3.5 [23].

## Supporting information

S1 Fig (Related to Fig 1) Dhh1 positively regulates autophagy under nitrogen-starvation conditions. (A) A replicate of the assay done in Fig 1C was conducted. S.E. and L.E. of the blot are shown. (B) Fba1–GFP (XLY320), Fba1–GFP *dhh1Δ* (XLY321), and Fba1–GFP *atg1Δ* (XLY322) cells were grown in YPD to mid-log phase (-N: 0 h) and then shifted to SD-N for 3 and 5 d. Cell lysates were prepared, subjected to SDS-PAGE, and analyzed by western blot. The image shown is from one blot. Some unrelated lanes were cropped. *atg1*, autophagy related 1; Fba1, fructose-1,6-biphosphate aldolase 1; GFP, green fluorescent protein; L.E., long exposure; SD-N, synthetic minimal medium lacking nitrogen; S.E., short exposure; YPD, yeast extract–peptone–dextrose.
(TIF)

S2 Fig (Related to Fig 2) Dhh1 promotes the translation of Atg1 and Atg13 during nitrogen starvation. (A) Analysis of structured regions in *ATG1*, *ATG2*, *VPS30/ATG6*, and *ATG13* mRNAs by SPARCS. The other *ATG* mRNAs tested for structured regions are shown in S1 Table. (B) Atg2–PA (XLY336) and Atg2–PA *dhh1Δ* (XLY337) cells were grown in YPD to mid-log phase (-N: 0 h) and then shifted to SD-N for 2, 4, and 6 h. Cell lysates were prepared, subjected to SDS-PAGE, and analyzed by western blot. Vma4 was a loading control. The 5′-UTR and 3′-UTR of *ATG2* in these strains were not changed. (C) WT (SEY6210), *dhh1Δ* (XLY301), and *vps30Δ* (JMY113) cells were grown in YPD to mid-log phase (-N: 0 h) and then shifted to SD-N for 6 and 24 h. Cell lysates were prepared, subjected to SDS-PAGE, and analyzed by western blot. (D) WT (SEY6210), *dhh1Δ* (XLY301), *atg1Δ* (XLY315), and *atg13Δ* (XLY352) cells were grown in YPD to mid-log phase (-N, 0 d) and then shifted to SD-N for 10 d. The indicated dilutions of cells were plated on YPD plates and grown for 2 d. Atg, autophagy-related; PA, protein A; SD-N, synthetic minimal medium lacking nitrogen; SPARCS, Structural Profile Assignment of RNA Coding Sequences; Vma4, vacuolar membrane ATPase 4; *VPS30*, vacuolar protein sorting 30; WT, wild type; YPD, yeast extract–peptone–dextrose.
(TIF)

S3 Fig (Related to Fig 2) DDX6 regulates ATG16L1 translation. (A) Analysis of structured regions in *ULK1* and *ATG16L1* mRNAs by SPARCS. (B) HEK293A WT or *DDX6* KO cells were incubated in amino acid–free medium for the indicated times. Proteins were analyzed through immunoblotting. (C) ATG16L1 protein level was quantified and normalized to ACTB. Relative ATG16L1 protein levels at the indicated time points were normalized to the zero (0, untreated) time point in the corresponding cell lines (WT, *n* = 5; *DDX6* KO, *n* = 4). (D) The *ATG16L1* mRNA level was quantified and normalized to *RPL7*. The relative *ATG16L1* mRNA levels at the indicated time points were normalized to the zero (0, untreated) time point in the corresponding cell lines (*n* = 3). (E) Basal level of ATG16L1 protein or mRNA relative to WT cells. Left panel: the ATG16L1 protein level was normalized to ACTB and then normalized to the levels from WT cells (*n* = 5). Right panel: the *ATG16L1* mRNA level was normalized to *RPL7* and then normalized to the levels from WT cells (*n* = 3). Data are presented as mean ± SEM; *p < 0.05. **p < 0.01. (Raw numerical values are shown in S1 Data). ACTB, actin beta; ATG16L1, autophagy related 16 like 1; DDX6, DEAD-box helicase 6; HEK293A, human embryonic kidney 293A; KO, knockout; NS, not significant in the Student *t* test; *RPL7*, ribosomal protein L7; SPARCS, Structural Profile Assignment of RNA Coding Sequences; *ULK1*, unc-51 like autophagy activating kinase 1; WT, wild type.
(TIF)

S4 Fig (Related to Fig 3) Dhh1 associates with *ATG1* and *ATG13* mRNAs during nitrogen starvation. (A) WT (SEY6210) and Dhh1–PA (XLY323) cells were grown in YPD to mid-log phase (-N, 0 h) and then shifted to SD-N for 2 h. The RNA immunoprecipitation assay was conducted and the data were analyzed as indicated in Fig 3B. *ALG9* mRNA was used as a negative control. Enrichment of the indicated 3′-UTR regions of *ATG* mRNAs was shown. *p < 0.05. **p < 0.01. (B) WT (SEY6210), *dhh1Δ* (XLY301), *PMP3p*–*ATG1* (XLY347), and *PMP3p*–*ATG1 dhh1Δ* (XLY348) cells were grown in YPD to mid-log phase (-N, 0 h) and then shifted to SD-N for 6 h. Cell lysates were prepared, subjected to SDS-PAGE, and analyzed by western blot. S.E., short exposure. L.E., long exposure. (C) *ATG1*–*ATG1*^*3′-UTR*^ (XLY316), *ATG1*–*ATG1*^*3′-UTR*^ *dhh1Δ* (XLY317), *ATG1*–*ATG7*^*3′-UTR*^ (XLY349), and *ATG1*–*ATG7*^*3′-UTR*^ *dhh1Δ* (XLY351) cells were grown in YPD to mid-log phase (-N, 0 h) and then shifted to SD-N for 6 h. Cell lysates were prepared, subjected to SDS-PAGE, and analyzed by western blot. (D) *ATG13*–*PA*–*ATG13*^*3′-UTR*^ (ZYY202), *ATG13*–*PA*–*ATG13*^*3′-UTR*^ *dhh1Δ* (ZYY203), *ATG13*–*PA*–*ATG7*^*3′-UTR*^ (ZYY213), and *ATG13*–*PA*–*ATG7*^*3′-UTR*^ *dhh1Δ* (ZYY214) cells were grown in YPD to mid-log phase (-N, 0 h) and then shifted to SD-N for 6 h. Cell lysates were prepared, subjected to SDS-PAGE, and analyzed by western blot. (Raw numerical values are shown in S1 Data). *ALG9*, asparagine-linked glycosylation 9; *ATG*, autophagy-related; L.E., long exposure; PA, protein A; *PMP3p*, plasma membrane proteolipid 3 promoter; SD-N, synthetic minimal medium lacking nitrogen; S.E., short exposure; WT, wild-type; YPD, yeast extract–peptone–dextrose.
(TIF)

S5 Fig (Related to Fig 4) The structured regions in the *ATG1* and *ATG13* ORFs are necessary for the translational regulation by Dhh1 after nitrogen starvation. (A) Analysis of structured regions in the mutated versions of *ATG1* and *ATG13* mRNAs by SPARCS. The corresponding mutated bases are indicated in Fig 4A. (B) The *dhh1Δ* strain with vectors expressing WT Dhh1–PA (XLY333), DEAA–PA (XLY334), or STAA–PA (XLY335) were grown in YPD to mid-log phase (-N: 0 h) and then shifted to SD-N for 6 h. Cell lysates were prepared, subjected to SDS-PAGE, and analyzed by western blot. (C) WT strain with empty vector (XLY329), the *dhh1Δ* strain with either empty vector (XLY331), or vectors expressing WT Dhh1–PA (XLY333), DEAA–PA (XLY334), or STAA–PA (XLY335) were grown in YPD to mid-log phase (-N: 0 h) and then shifted to SD-N for 24 h. Cell lysates were prepared, subjected to SDS-PAGE, and analyzed by western blot. *ATG*, autophagy-related; DEAA, Dhh1^D195A,E196A^; ORF, open reading frame; PA, protein A; SD-N, synthetic minimal medium lacking nitrogen; SPARCS, Structural Profile Assignment of RNA Coding Sequences; WT, wild-type; YPD, yeast extract–peptone–dextrose.
(TIF)

S6 Fig (Related to Fig 5) Dhh1 interacts with Eap1 under nitrogen-starvation conditions. (A) *EAP1–PA* (XLY344) and *ZEO1p–EAP1–PA* (ZYY207; *ZEO1* promoter, OE) cells were grown in YPD to mid-log phase (-N, 0 h) and then shifted to SD-N for 2 h. Cell lysates were prepared, subjected to SDS-PAGE, and analyzed by western blot. (B-C) Predictions of IDRs by IUPred2 and disordered binding regions by ANCHOR2 in Dhh1 (B) and Eap1 (C). Regions of the protein above the horizontal dashed line (scores = 0.5) are predicted to be disordered (red line) or to be disordered binding regions (blue line). (D) WT *DHH1–3HA* (ZYY208), *ZEO1p–EAP1–PA* WT *DHH1–3HA* (ZYY209), and *ZEO1p–EAP1–PA DHH1[1–425]–3HA* (XLY353) cells were grown in YPD to mid-log phase (-N, 0 h) and then shifted to SD-N for 2 h. The samples were collected and subjected to the protein–protein immunoprecipitation procedures described in the Materials and methods. The analysis of the samples by western blot is shown. (E) *DHH1–3HA* (ZYY208), *ZEO1p–EAP1–PA DHH1–3HA* (ZYY209; WT), and *ZEO1p–EAP1[1–270]–PA DHH1–3HA* (ZYY225) were cultured and subjected to procedures as indicated in (D). The analysis of the samples by western blot is shown. Eap1, eukaryotic translation initiation factor 4E–associated protein 1; IDR, intrinsically disordered region; L.E., long exposure; *PA*, protein A; SD-N, synthetic minimal medium lacking nitrogen; S.E., short exposure; WT, wild type; YPD, yeast extract–peptone–dextrose; *ZEO1p*, zeocin resistance 1 promoter.
(TIF)

S7 Fig (Related to Fig 6) Caf20 is not required for Atg1 translation and autophagy under nitrogen-starvation conditions. (A-B) Pgi1–GFP (XLY312) and Pgi1–GFP *caf20Δ* (XLY343) cells were grown in YPD to mid-log phase (-N: 0 h) and then shifted to SD-N for 6 or 24 h. Cell lysates were prepared, subjected to SDS-PAGE, and analyzed by western blot. Analysis of Atg1 protein levels and processing of Pgi1–GFP are shown in (A) and (B), respectively. Atg1, autophagy-related 1; Caf20, cap associated factor 20; GFP, green fluorescent protein; Pgi1, phosphoglucoisomerase 1; SD-N, synthetic minimal medium lacking nitrogen; YPD, yeast extract–peptone–dextrose.
(TIF)

S1 Table (Related to Fig 1) Yeast strains used in this study. (DOCX)

S2 Table (Related to Fig 2) Analysis of structured regions in *ATG* mRNAs within the core molecular machinery of autophagy by SPARCS. *ATG*, autophagy-related; SPARCS, Structural Profile Assignment of RNA Coding Sequences.
(DOCX)

S3 Table (Related to Fig 3) Primers for RT-qPCR analysis in yeast and HEK293A cells. HEK293A, human embryonic kidney 293A; RT-qPCR, quantitative reverse transcription PCR.
(DOCX)

S1 Data The numbers correspond to the indicated ratios for each individual experiment (#1–3) along with the average, SD or SE, and statistical significance value. WT or vehicle (DMSO)-only values were set to 1.0 (or to 100%), and other values were normalized to the WT where indicated. DMSO, dimethyl sulfoxide; WT, wild type.
(XLSX)

## Abbreviations

AID: auxin-inducible degron
*ALG9*: asparagine-linked glycosylation 9
Atg: autophagy related
ATG16L1: autophagy related 16 like 1
Caf20: cap associated factor 20
*CDK1*: cyclin dependent kinase 1
Dcp2: mRNA decapping 2
DDX6: DEAD-box helicase 6
DMSO: dimethyl sulfoxide
Eap1: EIF4E-associated protein 1
EIF4: eukaryotic translation initiation factor 4
EIF4EBP: EIF4E binding protein
*EZH2*: enhancer of zeste 2 polycomb repressive complex 2 subunit
Fba1: fructose-1,6-biphosphate aldolase 1
GFP: green fluorescent protein
HEK293A: human embryonic kidney 293A
IAA: indole-3-acetic acid
IDR: intrinsically disordered region
IgG: immunoglobulin G
IP: immunoprecipitation
*KLF4*: Kruppel like factor 4
L.E.: long exposure
MTORC1: mechanistic target of rapamycin kinase complex 1
NS: not significant
ORF: open reading frame
PA: protein A
Pgi1: phosphoglucoisomerase 1
*PMP3*: plasma membrane proteolipid 3
RT-qPCR: quantitative reverse transcription PCR
SPARCS: Structural Profile Assignment of RNA Coding Sequences
TOR: target of rapamycin
*ULK1*: unc-51 like autophagy activating kinase 1
*VPS30*: vacuolar protein sorting 30
WT: wild type
Xrn1: exoribonuclease 1
*ZEO1p*: zeocin resistance 1 promoter
