# Supplementary figures and images for: Dhh1 promotes autophagy-related protein translation during nitrogen starvation
Source: PLoS Biol. 2019 Apr 11;17(4):e3000219. doi: 10.1371/journal.pbio.3000219 (PMC6459490; doi:10.1371/journal.pbio.3000219)

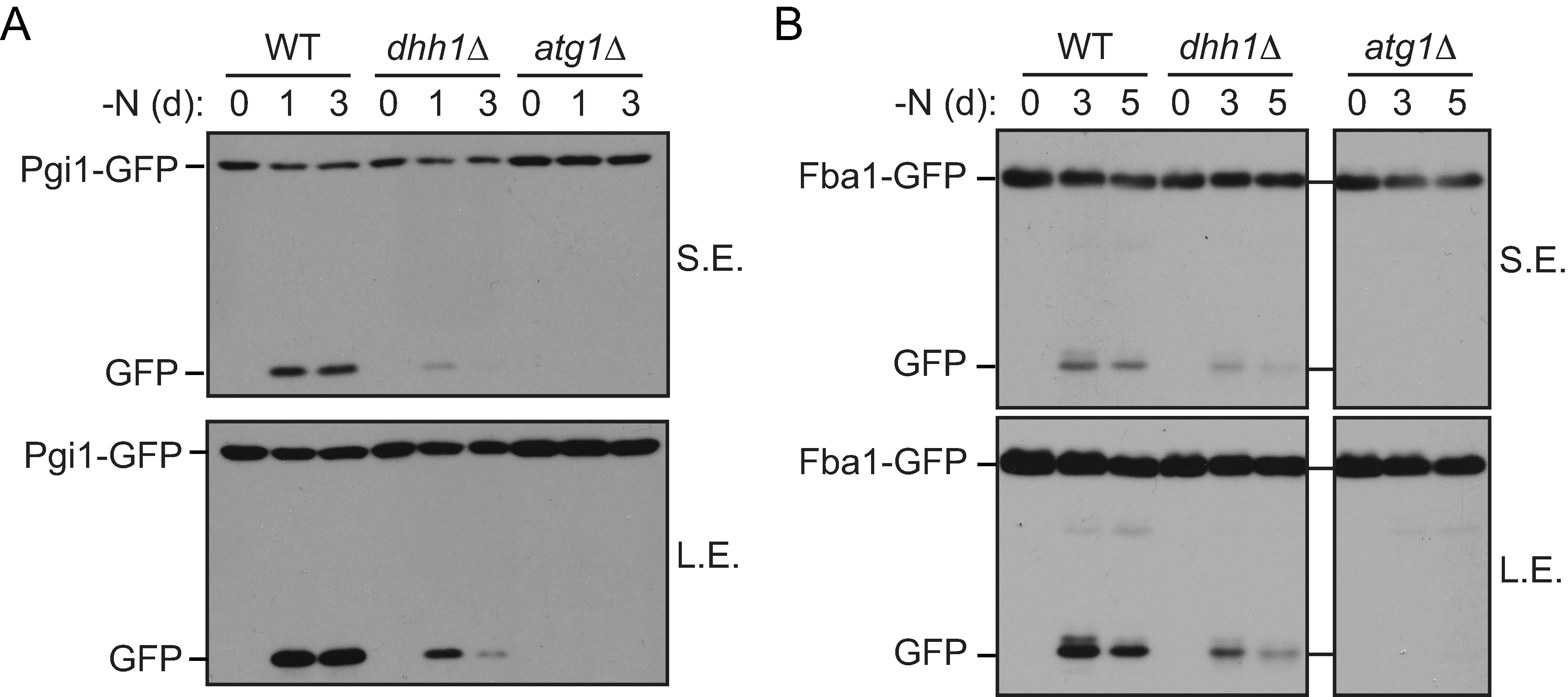

Supplement: S1 Fig — (A) A replicate of the assay done in Fig 1C was conducted. S.E. and L.E. of the blot are shown. (B) Fba1–GFP (XLY320), Fba1–GFP dhh1Δ (XLY321), and Fba1–GFP atg1Δ (XLY322) cells were grown in YPD to mid-log phase (-N: 0 h) and then shifted to SD-N for 3 and 5 d. Cell lysates were prepared, subjected to SDS-PAGE, and analyzed by western blot. The image shown is from one blot. Some unrelated lanes were cropped. atg1, autophagy related 1; Fba1, fructose-1,6-biphosphate aldolase 1; GFP, green fluorescent protein; L.E., long exposure; SD-N, synthetic minimal medium lacking nitrogen; S.E., short exposure; YPD, yeast extract–peptone–dextrose. (TIF) [file pbio.3000219.s001.tif]

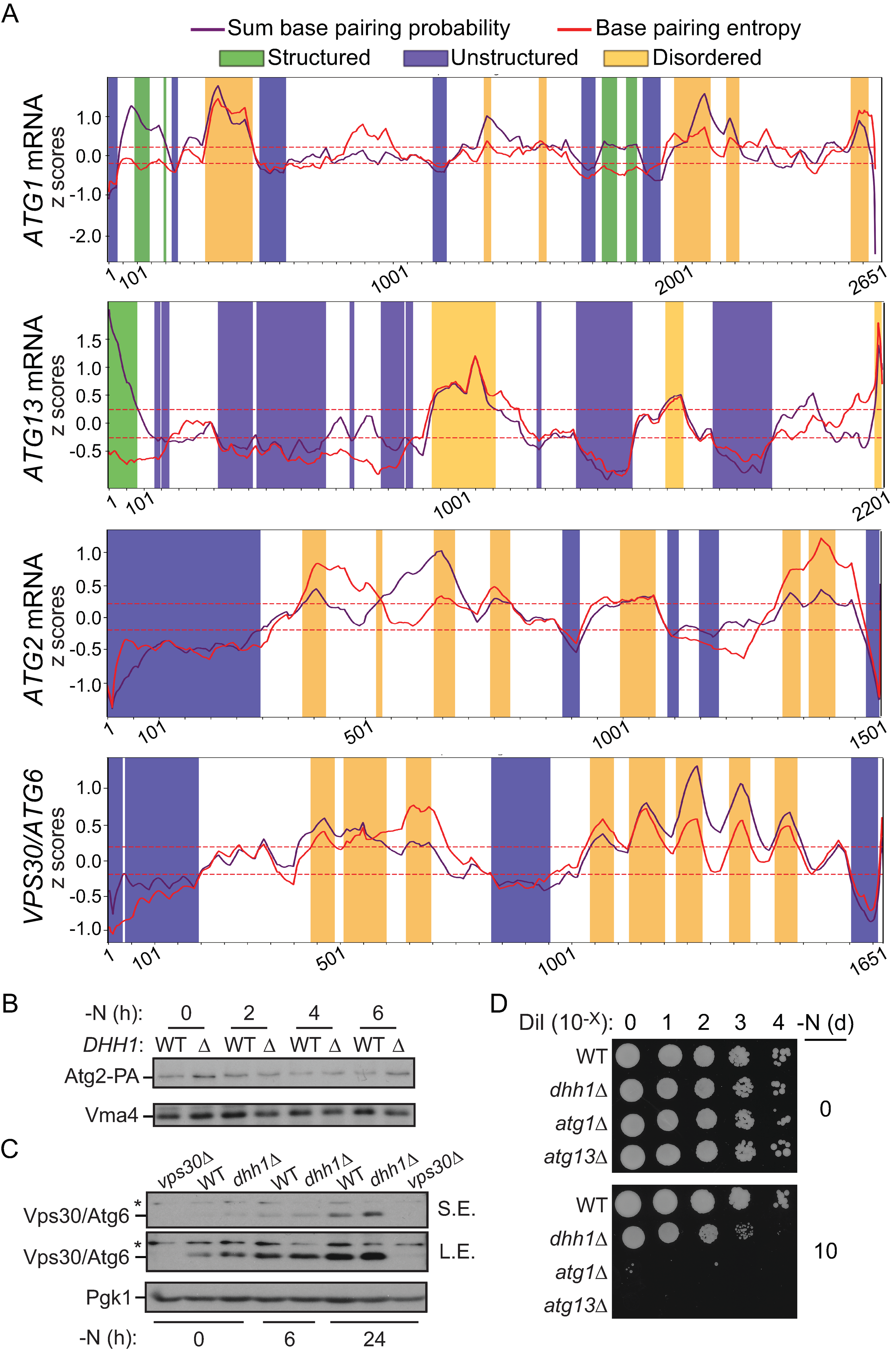

Supplement: S2 Fig — (A) Analysis of structured regions in ATG1, ATG2, VPS30/ATG6, and ATG13 mRNAs by SPARCS. The other ATG mRNAs tested for structured regions are shown in S1 Table. (B) Atg2–PA (XLY336) and Atg2–PA dhh1Δ (XLY337) cells were grown in YPD to mid-log phase (-N: 0 h) and then shifted to SD-N for 2, 4, and 6 h. Cell lysates were prepared, subjected to SDS-PAGE, and analyzed by western blot. Vma4 was a loading control. The 5′-UTR and 3′-UTR of ATG2 in these strains were not changed. (C) WT (SEY6210), dhh1Δ (XLY301), and vps30Δ (JMY113) cells were grown in YPD to mid-log phase (-N: 0 h) and then shifted to SD-N for 6 and 24 h. Cell lysates were prepared, subjected to SDS-PAGE, and analyzed by western blot. (D) WT (SEY6210), dhh1Δ (XLY301), atg1Δ (XLY315), and atg13Δ (XLY352) cells were grown in YPD to mid-log phase (-N, 0 d) and then shifted to SD-N for 10 d. The indicated dilutions of cells were plated on YPD plates and grown for 2 d. Atg, autophagy-related; PA, protein A; SD-N, synthetic minimal medium lacking nitrogen; SPARCS, Structural Profile Assignment of RNA Coding Sequences; Vma4, vacuolar membrane ATPase 4; VPS30, vacuolar protein sorting 30; WT, wild type; YPD, yeast extract–peptone–dextrose. (TIF) [file pbio.3000219.s002.tif]

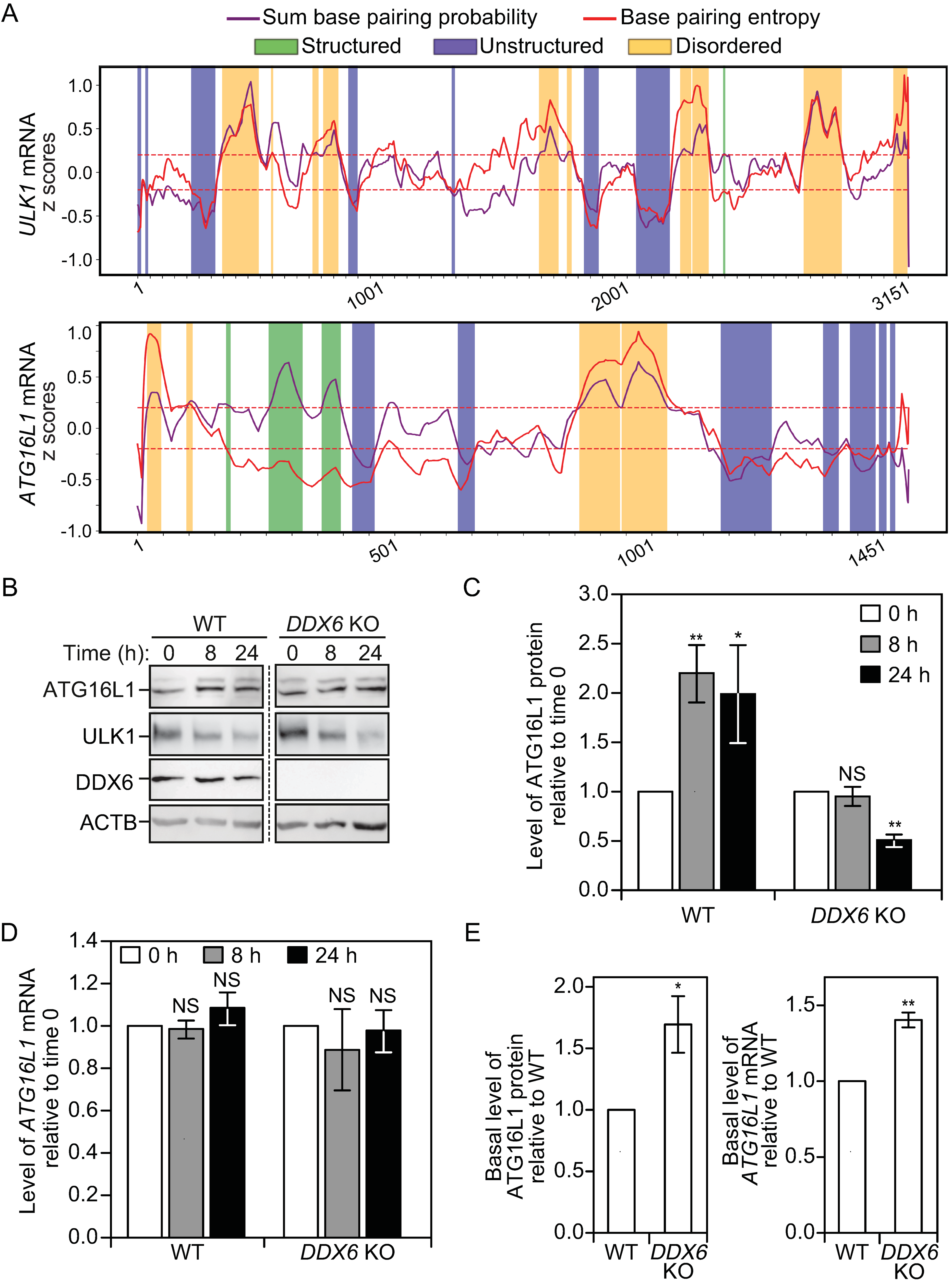

Supplement: S3 Fig — (A) Analysis of structured regions in ULK1 and ATG16L1 mRNAs by SPARCS. (B) HEK293A WT or DDX6 KO cells were incubated in amino acid–free medium for the indicated times. Proteins were analyzed through immunoblotting. (C) ATG16L1 protein level was quantified and normalized to ACTB. Relative ATG16L1 protein levels at the indicated time points were normalized to the zero (0, untreated) time point in the corresponding cell lines (WT, n = 5; DDX6 KO, n = 4). (D) The ATG16L1 mRNA level was quantified and normalized to RPL7. The relative ATG16L1 mRNA levels at the indicated time points were normalized to the zero (0, untreated) time point in the corresponding cell lines (n = 3). (E) Basal level of ATG16L1 protein or mRNA relative to WT cells. Left panel: the ATG16L1 protein level was normalized to ACTB and then normalized to the levels from WT cells (n = 5). Right panel: the ATG16L1 mRNA level was normalized to RPL7 and then normalized to the levels from WT cells (n = 3). Data are presented as mean ± SEM; *p < 0.05. **p < 0.01. (Raw numerical values are shown in S1 Data). ACTB, actin beta; ATG16L1, autophagy related 16 like 1; DDX6, DEAD-box helicase 6; HEK293A, human embryonic kidney 293A; KO, knockout; NS, not significant in the Student t test; RPL7, ribosomal protein L7; SPARCS, Structural Profile Assignment of RNA Coding Sequences; ULK1, unc-51 like autophagy activating kinase 1; WT, wild type. (TIF) [file pbio.3000219.s003.tif]

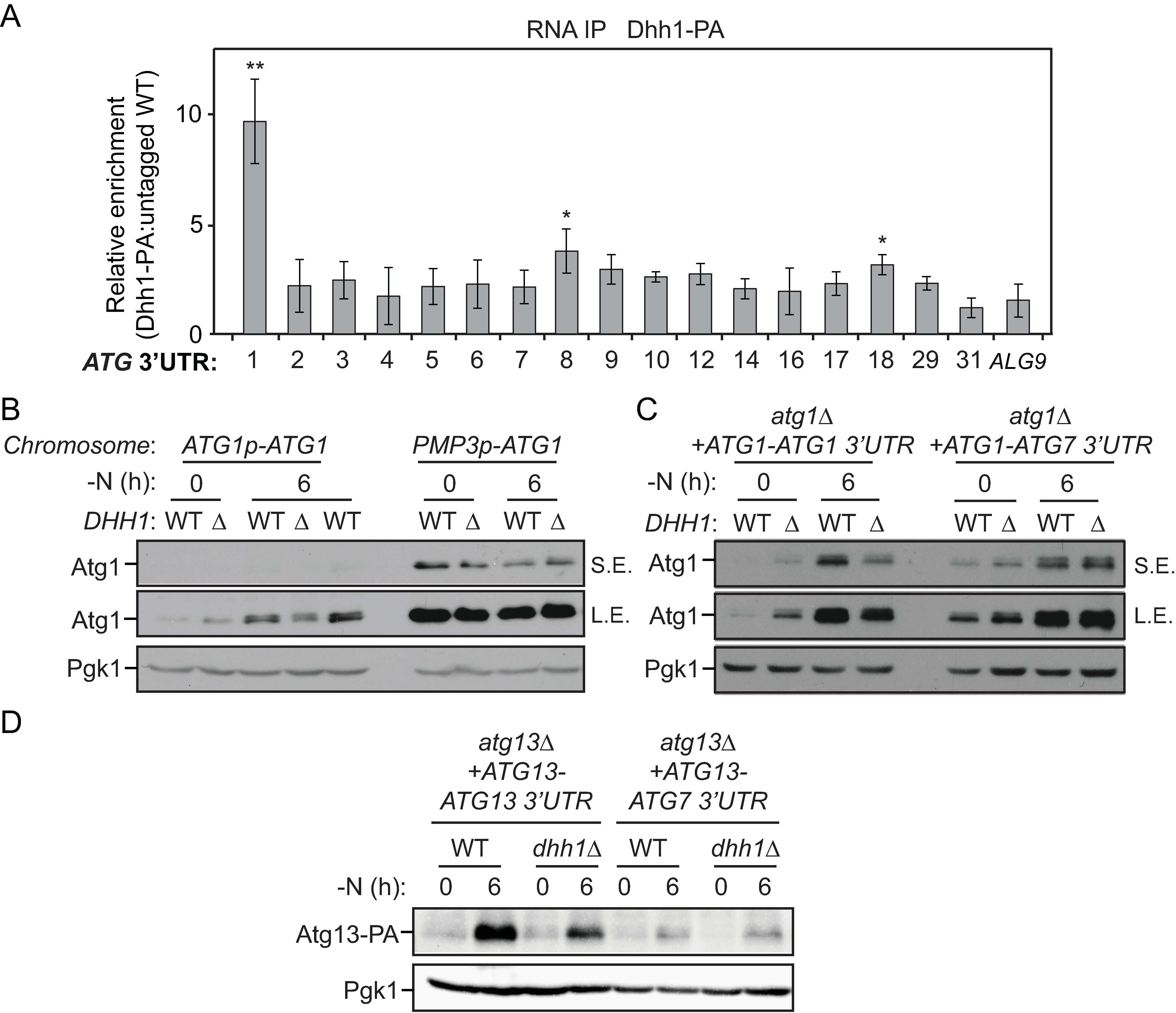

Supplement: S4 Fig — (A) WT (SEY6210) and Dhh1–PA (XLY323) cells were grown in YPD to mid-log phase (-N, 0 h) and then shifted to SD-N for 2 h. The RNA immunoprecipitation assay was conducted and the data were analyzed as indicated in Fig 3B. ALG9 mRNA was used as a negative control. Enrichment of the indicated 3′-UTR regions of ATG mRNAs was shown. *p < 0.05. **p < 0.01. (B) WT (SEY6210), dhh1Δ (XLY301), PMP3p–ATG1 (XLY347), and PMP3p–ATG1 dhh1Δ (XLY348) cells were grown in YPD to mid-log phase (-N, 0 h) and then shifted to SD-N for 6 h. Cell lysates were prepared, subjected to SDS-PAGE, and analyzed by western blot. S.E., short exposure. L.E., long exposure. (C) ATG1–ATG13′-UTR (XLY316), ATG1–ATG13′-UTR dhh1Δ (XLY317), ATG1–ATG73′-UTR (XLY349), and ATG1–ATG73′-UTR dhh1Δ (XLY351) cells were grown in YPD to mid-log phase (-N, 0 h) and then shifted to SD-N for 6 h. Cell lysates were prepared, subjected to SDS-PAGE, and analyzed by western blot. (D) ATG13–PA–ATG133′-UTR (ZYY202), ATG13–PA–ATG133′-UTR dhh1Δ (ZYY203), ATG13–PA–ATG73′-UTR (ZYY213), and ATG13–PA–ATG73′-UTR dhh1Δ (ZYY214) cells were grown in YPD to mid-log phase (-N, 0 h) and then shifted to SD-N for 6 h. Cell lysates were prepared, subjected to SDS-PAGE, and analyzed by western blot. (Raw numerical values are shown in S1 Data). ALG9, asparagine-linked glycosylation 9; ATG, autophagy-related; L.E., long exposure; PA, protein A; PMP3p, plasma membrane proteolipid 3 promoter; SD-N, synthetic minimal medium lacking nitrogen; S.E., short exposure; WT, wild-type; YPD, yeast extract–peptone–dextrose. (TIF) [file pbio.3000219.s004.tif]

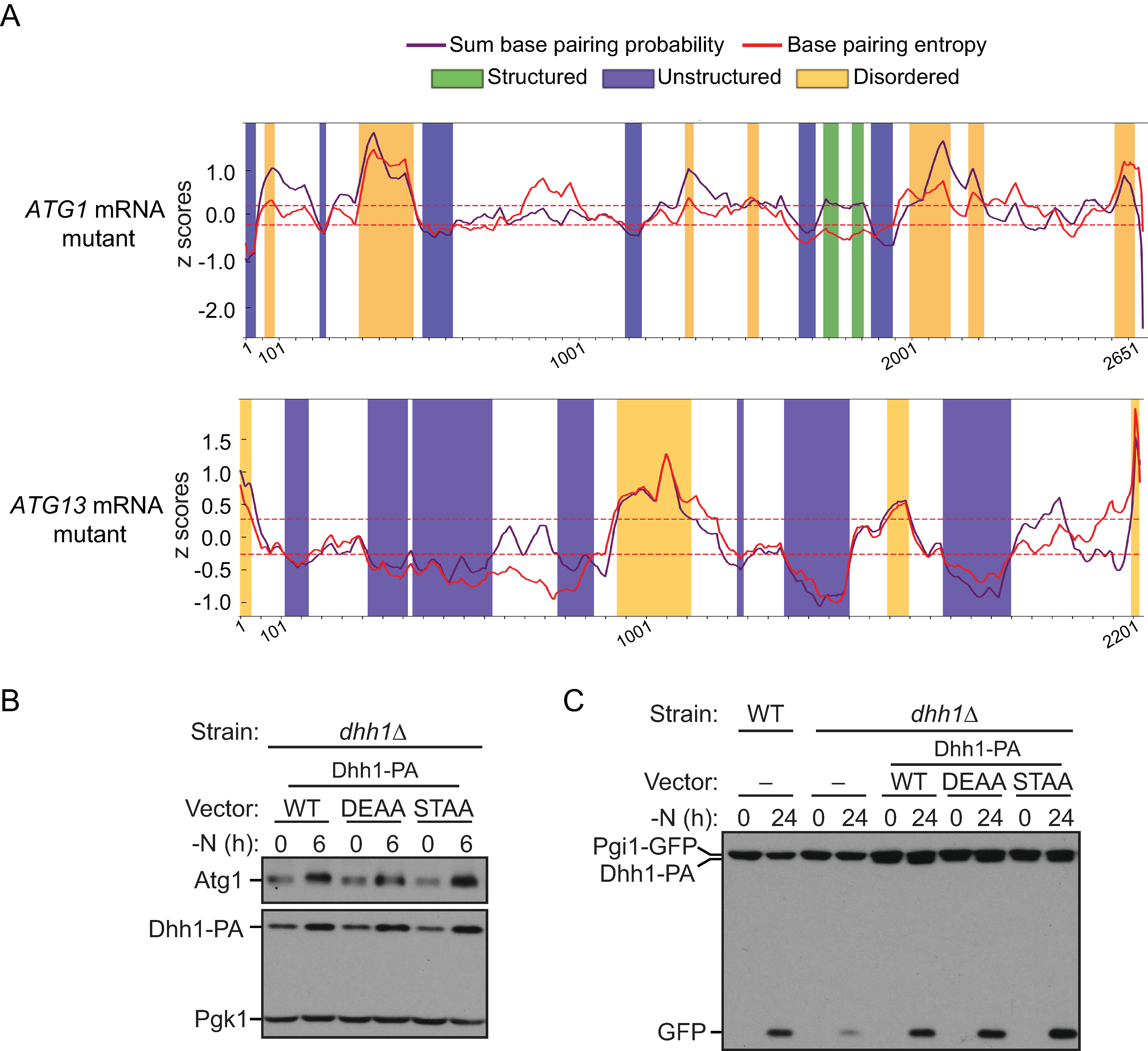

Supplement: S5 Fig — (A) Analysis of structured regions in the mutated versions of ATG1 and ATG13 mRNAs by SPARCS. The corresponding mutated bases are indicated in Fig 4A. (B) The dhh1Δ strain with vectors expressing WT Dhh1–PA (XLY333), DEAA–PA (XLY334), or STAA–PA (XLY335) were grown in YPD to mid-log phase (-N: 0 h) and then shifted to SD-N for 6 h. Cell lysates were prepared, subjected to SDS-PAGE, and analyzed by western blot. (C) WT strain with empty vector (XLY329), the dhh1Δ strain with either empty vector (XLY331), or vectors expressing WT Dhh1–PA (XLY333), DEAA–PA (XLY334), or STAA–PA (XLY335) were grown in YPD to mid-log phase (-N: 0 h) and then shifted to SD-N for 24 h. Cell lysates were prepared, subjected to SDS-PAGE, and analyzed by western blot. ATG, autophagy-related; DEAA, Dhh1D195A,E196A; ORF, open reading frame; PA, protein A; SD-N, synthetic minimal medium lacking nitrogen; SPARCS, Structural Profile Assignment of RNA Coding Sequences; WT, wild-type; YPD, yeast extract–peptone–dextrose. (TIF) [file pbio.3000219.s005.tif]

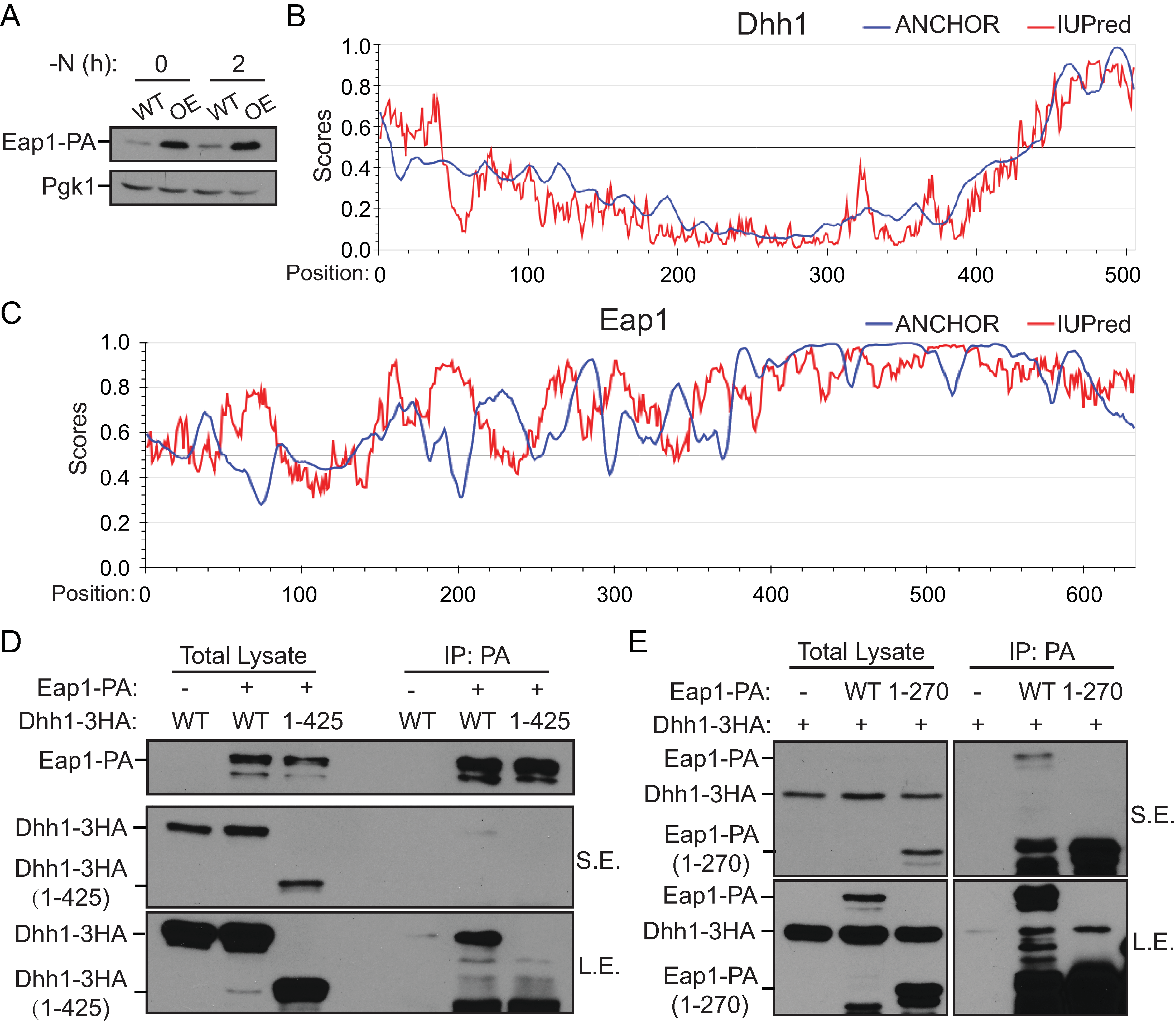

Supplement: S6 Fig — (A) EAP1–PA (XLY344) and ZEO1p–EAP1–PA (ZYY207; ZEO1 promoter, OE) cells were grown in YPD to mid-log phase (-N, 0 h) and then shifted to SD-N for 2 h. Cell lysates were prepared, subjected to SDS-PAGE, and analyzed by western blot. (B-C) Predictions of IDRs by IUPred2 and disordered binding regions by ANCHOR2 in Dhh1 (B) and Eap1 (C). Regions of the protein above the horizontal dashed line (scores = 0.5) are predicted to be disordered (red line) or to be disordered binding regions (blue line). (D) WT DHH1–3HA (ZYY208), ZEO1p–EAP1–PA WT DHH1–3HA (ZYY209), and ZEO1p–EAP1–PA DHH1[1–425]–3HA (XLY353) cells were grown in YPD to mid-log phase (-N, 0 h) and then shifted to SD-N for 2 h. The samples were collected and subjected to the protein–protein immunoprecipitation procedures described in the Materials and methods. The analysis of the samples by western blot is shown. (E) DHH1–3HA (ZYY208), ZEO1p–EAP1–PA DHH1–3HA (ZYY209; WT), and ZEO1p–EAP1[1–270]–PA DHH1–3HA (ZYY225) were cultured and subjected to procedures as indicated in (D). The analysis of the samples by western blot is shown. Eap1, eukaryotic translation initiation factor 4E–associated protein 1; IDR, intrinsically disordered region; L.E., long exposure; PA, protein A; SD-N, synthetic minimal medium lacking nitrogen; S.E., short exposure; WT, wild type; YPD, yeast extract–peptone–dextrose; ZEO1p, zeocin resistance 1 promoter. (TIF) [file pbio.3000219.s006.tif]

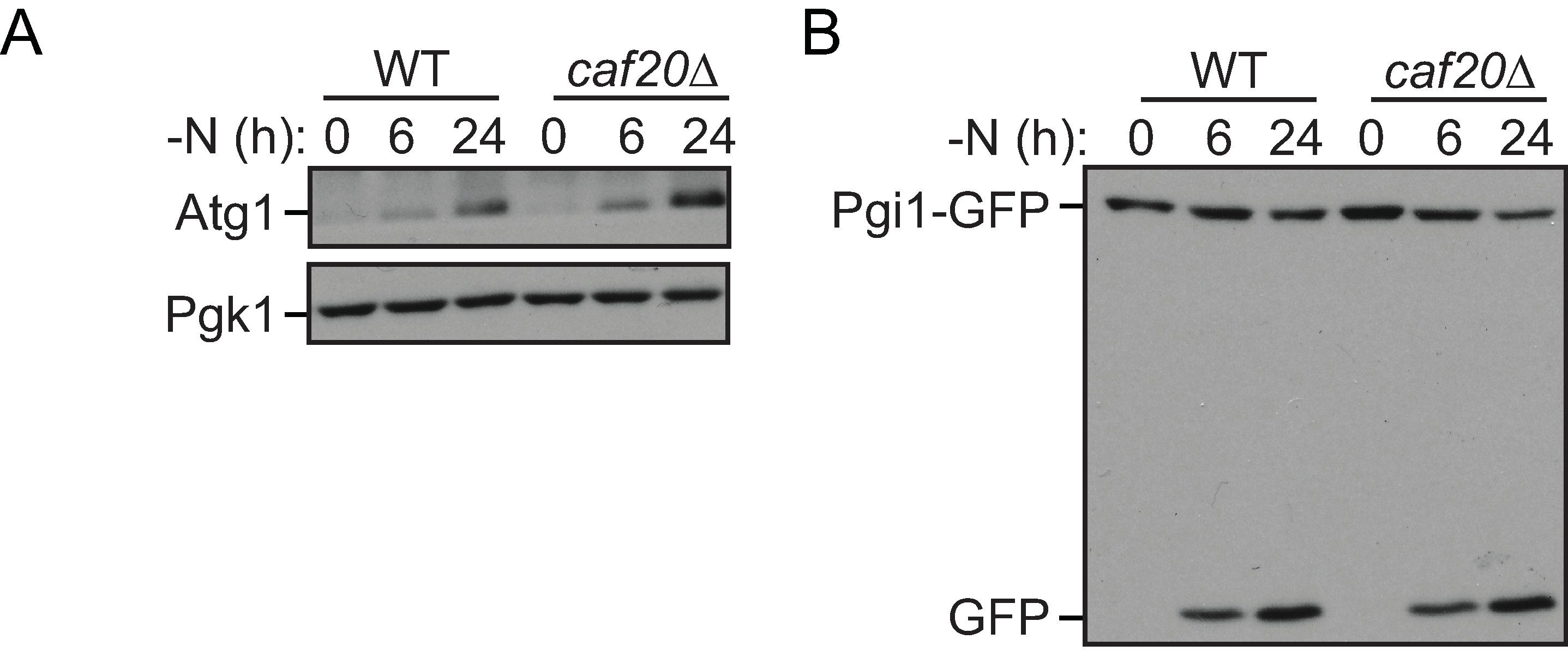

Supplement: S7 Fig — (A-B) Pgi1–GFP (XLY312) and Pgi1–GFP caf20Δ (XLY343) cells were grown in YPD to mid-log phase (-N: 0 h) and then shifted to SD-N for 6 or 24 h. Cell lysates were prepared, subjected to SDS-PAGE, and analyzed by western blot. Analysis of Atg1 protein levels and processing of Pgi1–GFP are shown in (A) and (B), respectively. Atg1, autophagy-related 1; Caf20, cap associated factor 20; GFP, green fluorescent protein; Pgi1, phosphoglucoisomerase 1; SD-N, synthetic minimal medium lacking nitrogen; YPD, yeast extract–peptone–dextrose. (TIF) [file pbio.3000219.s007.tif]
