# Supplementary material for: Dhh1 promotes autophagy-related protein translation during nitrogen starvation
Source: PLoS Biol. 2019 Apr 11;17(4):e3000219. doi: 10.1371/journal.pbio.3000219 (PMC6459490; doi:10.1371/journal.pbio.3000219)
Supplement: S1 Table — (DOCX) [file pbio.3000219.s008.docx]

**S1 Table (related to Figure 1): Yeast strains used in this study.**

| **Name** | **Genotype** | **Reference** |
| --- | --- | --- |
| BY4742 | MATα *his3∆1 leu2∆0 lys2∆0 ura3∆0* | Invitrogen |
| JMY113 | WLY176 *vps30∆::KANMX6* | This study |
| JMY114 | WLY176 *atg13∆::KANMX6* | This study |
| SEY6210 | MATα *leu2-3,112 ura3-52 his3-∆200 trp1-∆901 suc2-∆9 lys2-801 GAL* | [1] |
| WLY176 | SEY6210 *pho13∆ pho8∆60* | [2] |
| XLY301 | SEY6210 *dhh1∆::KANMX6* | This study |
| XLY306 | BY4742 *PGI1-GFP::HIS3* | This study |
| XLY307 | BY4742 *PGI1-GFP::HIS3 atg1∆::URA3* | This study |
| XLY308 | BY4742 *PGI1-GFP::HIS3 dhh1∆::URA3* | This study |
| XLY310 | BY4742 *PGI1-GFP::HIS3 eap1∆::KANMX6* | This study |
| XLY312 | SEY6210 *PGI1-GFP:TRP1* | This study |
| XLY314 | SEY6210 *PGI1-GFP:TRP1 dhh1∆::KANMX6* | This study |
| XLY316 | SEY6210 *atg1∆::HIS3 pRS-ATG1(406)::URA3* | This study |
| XLY317 | SEY6210 *atg1∆::HIS3 pRS-ATG1(406)::URA3 dhh1∆::KANMX6* | This study |
| XLY318 | SEY6210 *atg1∆::HIS3 pRS-ATG1^mut^(406)::URA3* | This study |
| XLY319 | SEY6210 *atg1∆::HIS3 pRS-ATG1^mut^(406)::URA3 dhh1∆::KANMX6* | This study |
| XLY320 | BY4742 *Fba1-GFP::HIS3* | This study |
| XLY321 | BY4742 *Fba1-GFP::HIS3 dhh1∆::URA3* | This study |
| XLY322 | BY4742 *Fba1-GFP::HIS3 atg1∆::URA3* | This study |
| XLY323 | SEY6210 *DHH1-PA::HIS3* | This study |
| XLY324 | SEY6210 *ATG1-ADH1 3'UTR::TRP1* | This study |
| XLY325 | SEY6210 *ATG1-ADH1 3'UTR::TRP1 dhh1∆::KANMX6* | This study |
| XLY326 | SEY6210 *ATG1-ADH1 3'UTR::TRP1 DHH1-PA::HIS3* | This study |
| XLY327 | SEY6210 *PGI1-GFP:TRP1 DHH1-PA::HIS3* | This study |
| XLY328 | SEY6210 *PGI1-GFP:TRP1* *DHH1(H395A,R396A)-PA::HIS3* | This study |
| XLY329 | SEY6210 *PGI1-GFP:TRP1 pRS(405)::LEU2* | This study |
| XLY331 | SEY6210 *PGI1-GFP:TRP1 dhh1∆::KANMX6 pRS(405)::LEU2* | This study |
| XLY333 | SEY6210 *PGI1-GFP:TRP1 dhh1∆::KANMX6 pRS-DHH1-PA(405)::LEU2* | This study |
| XLY334 | SEY6210 *PGI1-GFP:TRP1 dhh1∆::KANMX6 pRS-DHH1(D195A,E196A)-PA(405)::LEU2* | This study |
| XLY335 | SEY6210 *PGI1-GFP:TRP1 dhh1∆::KANMX6 pRS-DHH1(S226A,T228A)-PA(405)::LEU2* | This study |
| XLY336 | WLY176 *ATG2-PA* | This study |
| XLY337 | WLY176 *ATG2-PA dhh1∆::URA3* | This study |
| XLY338 | SEY6210 *pNHK53 (ADH1p-OsTIR1-9MYC)::URA3* | This study |
| XLY339 | SEY6210 *pNHK53 (ADH1p-OsTIR1-9MYC)::URA3 pGFP-ATG8 (405)::LEU2* | This study |
| XLY340 | XLY338 *DHH1-AID-9MYC::KANMX6* | This study |
| XLY341 | XLY339 *DHH1-AID-9MYC::KANMX6* | This study |
| XLY342 | SEY6210 *PGI1-GFP:TRP1 dhh1∆::KANMX6 pRS-DHH1(D195A,E196A,S226A,T228A)-PA(405)::LEU2* | This study |
| XLY343 | SEY6210 *PGI1-GFP:TRP1 caf20∆::KANMX6* | This study |
| XLY344 | SEY6210 *EAP1-PA::KANMX6* | This study |
| XLY345 | SEY6210 *DHH1-3HA::TRP1 EAP1-PA::KANMX6* | This study |
| XLY346 | SEY6210 *HIS3::ZEO1p-EAP1-GFP::TRP1* | This study |
| XLY347 | SEY6210 *HIS3::PMP3p-ATG1* | This study |
| XLY348 | SEY6210 *HIS3::PMP3p-ATG1 dhh1∆::KANMX6* | This study |
| XLY349 | SEY6210 *atg1∆::HIS3 pRS-ATG1-ATG7^3'UTR^(406)::URA3* | This study |
| XLY351 | SEY6210 *atg1∆::HIS3 pRS-ATG1-ATG7^3'UTR^(406)::URA3 dhh1∆::KANMX6* | This study |
| XLY352 | SEY6210 *atg13∆::HIS3* | This study |
| XLY353 | SEY6210 *HIS3::ZEO1p-EAP1-PA::KAN DHH1(1-425)-3HA::TRP1* | This study |
| YZY256 | SEY6210 *HIS3::ZEO1p-EAP1-GFP::TRP1 DHH1-13MYC::KANMX6* | This study |
| ZYY101 | WLY176 *pRS405-GFP-ATG8::LEU2* | [3] |
| ZYY201 | WLY176 *pRS405-GFP-ATG8::LEU2 dhh1∆::KANMX6* | This study |
| ZYY202 | JMY114 *pRS406-ATG13-PA::URA3* | This study |
| ZYY203 | JMY114 *pRS406-ATG13-PA::URA3 dhh1∆::HIS3* | This study |
| ZYY204 | JMY114 *pRS406-ATG13-PA::URA3 eap1∆::HIS3* | This study |
| ZYY205 | JMY114 *pRS406-ATG13^mut^-PA::URA3* | This study |
| ZYY206 | JMY114 *pRS406-ATG13^mut^-PA::URA3 dhh1∆::HIS3* | This study |
| ZYY207 | SEY6210 *HIS3::ZEO1p-EAP1-PA::KANMX6* | This study |
| ZYY208 | SEY6210 *DHH1-3HA::TRP1* | This study |
| ZYY209 | SEY6210 *HIS3::ZEO1p-EAP1-PA::KAN DHH1-3HA::TRP1* | This study |
| ZYY210 | XLY338 *EAP1-AID-9MYC::HIS3* | This study |
| ZYY211 | ZYY202 *EAP1(∆271-632)-GFP::TRP1* | This study |
| ZYY212 | ZYY202 *EAP1(∆441-632)-GFP::TRP1* | This study |
| ZYY213 | JMY114 *pRS406-ATG13-PA-ATG7^3'UTR^::URA3* | This study |
| ZYY214 | ZYY213 *dhh1∆::HIS3* | This study |
| ZYY215 | ZYY202 *EAP1-GFP::TRP1* | This study |
| ZYY225 | SEY6210 *HIS3::ZEO1p-EAP1-PA(1-270)::KAN DHH1-3HA::TRP1* | This study |

The yeast strain names and genotypes are indicated. The parental strain used for subsequent genetic modification is indicated as the first part of the genotype.

**Supplemental References**

1. Robinson JS, Klionsky DJ, Banta LM, Emr SD. Protein sorting in Saccharomyces cerevisiae: isolation of mutants defective in the delivery and processing of multiple vacuolar hydrolases. Mol Cell Biol. 1988;8: 4936-4948.

2. Kanki T, Wang K, Baba M, Bartholomew CR, Lynch-Day MA, Du Z, et al. A genomic screen for yeast mutants defective in selective mitochondria autophagy. Mol Biol Cell. 2009;20: 4730-4738. doi: 10.1091/mbc.E09-03-0225.

3. Yao Z, Delorme-Axford E, Backues SK, Klionsky DJ. Atg41/Icy2 regulates autophagosome formation. Autophagy. 2015;11: 2288-2299. doi: 10.1080/15548627.2015.1107692.
