# Supplementary material for: Dhh1 promotes autophagy-related protein translation during nitrogen starvation
Source: PLoS Biol. 2019 Apr 11;17(4):e3000219. doi: 10.1371/journal.pbio.3000219 (PMC6459490; doi:10.1371/journal.pbio.3000219)
Supplement: S2 Table — ATG, autophagy-related; SPARCS, Structural Profile Assignment of RNA Coding Sequences. (DOCX) [file pbio.3000219.s009.docx]

**S2 Table (related to Figure 2). Analysis of structured regions in *ATG* mRNAs within the core molecular machinery of autophagy by SPARCS**

| **mRNA** | **Structured region** | **Unstructured region** | **Disordered region** |
| --- | --- | --- | --- |
| *ATG1* | [91, 145], [194, 203], [1733, 1787], [1820, 1858] | [1, 32], [222, 244], [530, 624], [1140, 1189], [1662, 1711], [1878, 1941] | [339, 506], [1319, 1345], [1512, 1539], [1988, 2116], [2171, 2217], [2608, 2671] |
| *ATG2 [1-1500]* |  | [1, 297], [883, 917], [1087, 1109], [1148, 1187], [1472, 1498] | [378, 424], [522, 534], [634, 675], [743, 782], [995, 1064], [1310, 1345], [1361, 1413] |
| *ATG3* |  | [13, 106], [113, 172], [200, 230], [393, 414] | [519, 661], [737, 758], [801, 884], [914, 931] |
| *ATG4* | [1421, 1443] | [107, 134], [141, 205], [494, 523], [579, 598], [680, 705], [879, 1008] | [16, 37], [1187, 1321] |
| *ATG5* | [256, 271], [330, 349], [687, 696] | [15, 119], [375, 509], [713, 764], [768, 815], [823, 872] | [545, 593] |
| *VPS30 (ATG6)* |  | [1, 32], [37, 196], [827, 955], [1604, 1662] | [437, 489], [508, 602], [643, 698], [1040, 1092], [1125, 1203], [1226, 1284], [1341, 1387], [1439, 1489] |
| *ATG7* |  | [1, 43], [143, 154], [437, 612], [652, 684], [1048, 1071], [1414, 1453], [1839, 1890] | [1481, 1610], [1674, 1825] |
| *ATG8* |  |  | [21, 182] |
| *ATG9 [1-1500]* | [1271, 1304], [1348, 1368] | [1, 103], [117, 134], [443, 490], [787, 830], [1040, 1130], [1465, 1499] | [159, 343], [388, 406], [511, 537], [577, 651] |
| *ATG10* |  | [1, 60], [130, 189], [222, 259], [421, 434], [447, 472] | [490, 502] |
| *ATG12* |  | [1, 17], [359, 385], [390, 412], [445, 516], [521, 549] | [161, 318] |
| *ATG13* | [1, 82] | [131, 148], [151, 176], [315, 411], [424, 543], [545, 618], [784, 877], [884, 897], [1227, 1238], [1339, 1502], [1689, 1698], [1731, 1901] | [927, 1126], [1594, 1648], [2195, 2216] |
| *ATG14* |  | [75, 150], [177, 208], [825, 871] | [983, 1027] |
| *ATG16* |  | [1, 100] | [251, 270], [274, 335], [417, 426] |
| *ATG17* |  | [1, 23], [341, 394], [659, 716], [1036, 1045] | [428, 452], [488, 559], [872, 910], [1237, 1253] |
| *ATG18* | [385, 422] | [6, 187], [222, 263], [450, 471], [630, 643], [1076, 1114], [1467, 1491] | [661, 825], [1195, 1273], [1300, 1318] |
| *ATG29* |  | [35, 395] | [483, 556], [596, 624] |
| *ATG31* | [416, 516] | [1, 91] | [208, 287], [297, 330] |

The beginning and end points (nucleotide positions) of the predicted structured, unstructured and disordered regions are indicated in brackets.
