# Supplementary material for: Dhh1 promotes autophagy-related protein translation during nitrogen starvation
Source: PLoS Biol. 2019 Apr 11;17(4):e3000219. doi: 10.1371/journal.pbio.3000219 (PMC6459490; doi:10.1371/journal.pbio.3000219)
Supplement: S3 Table — HEK293A, human embryonic kidney 293A; RT-qPCR, quantitative reverse transcription PCR. (DOCX) [file pbio.3000219.s010.docx]

**S3 Table (related to Figure 3). Primers for RT-qPCR analysis in yeast and HEK293A cells**

| **Primer** | **Sequence** |
| --- | --- |
| *ATG1* -480 F | TTAACCGCTCGGCTCTGATTTC |
| *ATG1* -480 R | AAGCTCCTTTATGAGATGCTCGATTC |
| *ATG1* -290 F | TAGGCCGAGGTTAATTCTAGAACG |
| *ATG1* -290 R | ATAGTACTGTTCTCTGTTTCCCCAGA |
| *ATG1* 35 F | CTGTGAACCATAATCTAATGGCAAGTG |
| *ATG1* 35 R | TACTTCCTTTATGGCTACATGCTGAG |
| *ATG1* 800 F | GAGCTTCCAATCATTTGGAGTTATTC |
| *ATG1* 800 R | CTATTCTTTGGGCTGGATCAAATGTC |
| *ATG1* 2340 F | GGTAGTTCGGAAGAGCCAGTATAT |
| *ATG1* 2340 R | GTTGCATAAGCTAATTCACAGTTGTAC |
| *ATG1* 3'UTR F | GAGGCAGAAGATGAACCACCAAA |
| *ATG1* 3'UTR R | GTAAAGCATTTCGAGAGTAGCATAAC |
| *ATG2* 3'UTR F | GTACAAGTCCAATCGGACTGATTC |
| *ATG2* 3'UTR R | GCTTTCCCACCATTCCACGTCTG |
| *ATG3* 3‘UTR F | CGTTACGGGTAGACCAATACTTGA |
| *ATG3* 3’UTR R | ACCTGGCTTGCAGCTAATAGTG |
| *ATG4* 3‘UTR F | GAAGGAAACGGTAGGTATTCACAG |
| *ATG4* 3'UTR R | AACTGGATACCTCAGTTTTAGGGA |
| *ATG5* 3'UTR F | TTCCTTGGCATATGCTCCTGTATG |
| *ATG5* 3'UTR R | TATAACAGCTCTTAGAGCTCAGAG |
| *VPS30/ATG6* 3'UTR F | CCTTGAATGGACAACGGCTATGA |
| *VPS30/ATG6* 3'UTR R | CTTAGTTTCCGCTGATGGTCTTATC |
| *ATG7* 3'UTR F | AGCGGTTTGTCAGTCATAAAGCAG |
| *ATG7* 3'UTR R | GTGGCACCACAATATGTACCAATG |
| *ATG8* 3'UTR F | CATTTGGCAGGTAGTCTTTTATATG |
| *ATG8* 3'UTR R | CTTATACTGGAACAATAGATGGCT |
| *ATG9* 3'UTR F | ACAATGGTAATGGCATACTCAATAAG |
| *ATG9* 3'UTR R | GGATGATGTACACGACACAGTCTG |
| *ATG10* 3'UTR F | GCGATACATCATGTATAGTAGGTGAC |
| *ATG10* 3'UTR R | GCTTTCCTAGGTTAAGCTATGAATC |
| *ATG12* 3'UTR F | GGATGCAATTCAAGACTAATGATGAG |
| *ATG12* 3'UTR R | GTACGGGATTTTTGATCGACTGTAG |
| *ATG13* -10 F | AGCATGAGTCATGGTTGCCGAAG |
| *ATG13* -10 R | ACTTGATTCGGTGGAGCATATTAG |
| *ATG13* 1000 F | CAACACCAGGTCCAGACACAAC |
| *ATG13* 1000 R | GTTGAACAGGTCTCGATATTGGATAG |
| *ATG13* 3'UTR F | GACGAGGATGATCAAGATGATGATCTAG |
| *ATG13* 3'UTR R | TCTTTTCTTGCATATCTATTTCACCTT |
| *ATG14* 3'UTR F | TACTGGACCAGTACGATGTG |
| *ATG14* 3'UTR R | TGCAGGATGTCCTCTTTGTG |
| *ATG16* 3'UTR F | GAACATTCCCAGCTAGTTGCGAG |
| *ATG16* 3'UTR R | TGACGATTTGACAACTGATGCATC |
| *ATG17* 3'UTR F | GAGGAGAAAGGATGTGGCAAACAG |
| *ATG17* 3'UTR R | GCCAGATTGTCTCAGGAAGATAAG |
| *ATG29* 3'UTR F | GCCTAATGACAAATTGGATAAGGA |
| *ATG29* 3'UTR R | CAATCTGTCCATTAGCGCTTCTTC |
| *ATG31* 3'UTR F | GGACTCGGACATTGAGCTCGATG |
| *ATG31* 3'UTR R | GCTTTCATACGGAATTGGAGAGCA |
| *ALG9* F | CACGGATAGTGGCTTTGGTGAACAATTAC |
| *ALG9* R | TATGATTATCTGGCAGCAGGAAAGAACTTGGG |
| *HsATG16L1* F1 | AACGCTGTGCAGTTCAGTCC |
| *HsATG16L1* R1 | AGCTGCTAAGAGGTAAGATCCA |
| *HsATG16L1* F2 | TCTGGGACATTCGATCAGAGAG |
| *HsATG16L1* R2 | CCTTTCTGGGTTTAAGTCCAGG |
| *HsRPL7* F | GCTTATCTATGAAAAAGCAAAGCAC |
| *HsRPL7* R | CATTGATACCTCTGATTCTGATGAC |

The forward and reverse primers used for RT-PCR detection of the listed genes are indicated by “-F” and “-R”, respectively.
